# Role of Hydrogen Bonding in Crystal Structure and Luminescence Properties of Melem Hydrates

**DOI:** 10.1021/acsomega.5c01714

**Published:** 2025-04-15

**Authors:** Kaname Kanai, Taiki Yamazaki, Hiroki Kiuchi, Momoka Isobe, Yoriko Sonoda

**Affiliations:** † Department of Physics and Astronomy, Faculty of Science and Technology, Tokyo University of Science, 2641 Yamazaki, Noda, Chiba 278-8510, Japan; ‡ Research Institute for Advanced Electronics and Photonics, National Institute of Advanced Industrial Science and Technology (AIST), Higashi 1-1-1, 305-8565 Tsukuba, Ibaraki, Japan

## Abstract

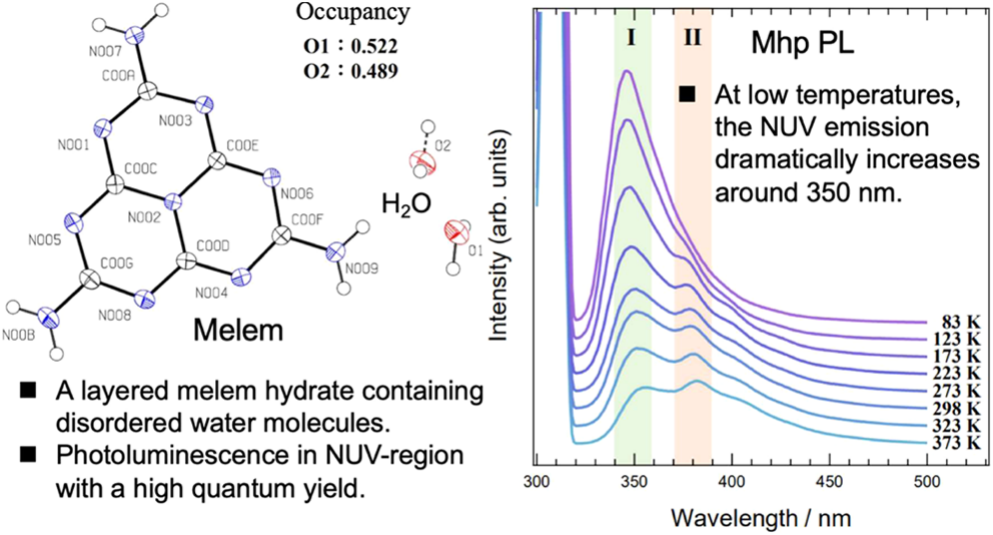

In recent years, carbon nitride (CN) compounds, such as g-C_3_N_4_ and melem, have attracted attention as new visible light-driven photocatalysts with a variety of functions, including water splitting, organic decomposition, and dark photocatalysis. The building unit of these materials is the heptazine ring, and molecules with this structure have attracted considerable attention as luminescent materials. Melem is an organic molecule with amino groups at the three termini of its heptazine ring. Melem exhibits near-UV (NUV) emission with high quantum yield via thermally activated delayed fluorescence (TADF). Materials exhibiting TADF can achieve highly efficient luminescence without the use of heavy metals, generating interest in their potential as luminescent materials for organic electroluminescent devices. Compared to materials that emit in the visible-light region, there are few reports on TADF materials such as melem that exhibit NUV emissions. Melem hydrate is easily obtained by hydrothermal treatment of melem. Unlike melem crystals, melem hydrate (Mh) has a porous structure because of a hydrogen-bond network formed between melem and water molecules. To date, only one type of Mh has been well-investigated. Mhs are expected to exhibit novel properties, such as photocatalysis, molecular adsorption, and highly efficient NUV emission. Mh also provides an opportunity to investigate how hydrogen bonds between the melem molecule and crystal water affect the TADF NUV emissions. This provides clues to the mechanism of the TADF action exhibited by other melem compounds. In this study, we focus on a new melem hydrate with a parallelogram shape, Mhp, first reported by Dai et al. in 2022. The crystal structure of Mhp reportedly differs from that of Mh; however, the Mhp crystal structure has not been determined to date, and its physical properties have not been investigated. Therefore, in this study, we reexamined the conditions for growing single crystals of Mhp and succeeded in growing samples that could be used to measure physical properties. We also determined its crystal structure and investigated the role in crystal formation of the hydrogen bonds between melem and water molecules. We evaluated the thermal behavior and optical properties and discussed their correlation with the crystal structure. Similar to melem, Mhp displayed NUV luminescence in its photoluminescence (PL) spectrum. This luminescence was found to have high quantum yield and delayed fluorescence. At low temperatures, the PL of Mhp dramatically increased at a wavelength of approximately 350 nm. This behavior was attributed to a significant change in the hydrogen-bond network between melem and water molecules in the Mhp crystal at low temperatures. We found that distortion of the melem molecule in the excited state at low temperatures was suppressed by its strong hydrogen bonds with water molecules. As a result, the displacement of the atomic nuclei of the atoms that make up the melem molecules in the excited state produced by light absorption is small, and in the de-excitation process, radiative transitions to low-energy vibrational levels are promoted. At the same time, nonradiative deactivation was suppressed, resulting in high fluorescence quantum efficiency. The results of this research provide deep insight into the role of hydrogen bonds in the optical properties of hydrate crystals that exhibit highly efficient luminescence, including TADF.

## 1 Introduction

In recent years, carbon nitride (CN) materials have attracted attention for their simple and inexpensive synthesis methods, metal-free composition, and photocatalytic activity in the visible light region. Molecules with a heptazine skeleton, which is a building unit of g-C_3_N_4_, are characterized by their rigidity and excellent atmospheric stability. Some molecules with heptazine frameworks have reportedly exhibited thermally activated delayed fluorescence (TADF) and have been actively investigated as light-emitting materials for organic electroluminescent devices such as organic light-emitting diodes.^1−3^ Recently, TADF, which can achieve highly efficient luminescence without costly, environmentally hazardous molecules containing heavy atoms, has attracted much attention, and molecules displaying TADF have been vigorously sought.^4^ The optical properties of these molecules are generally controlled by changing the type of functional group at the end of the heptazine frameworks.

**Figure 1:**
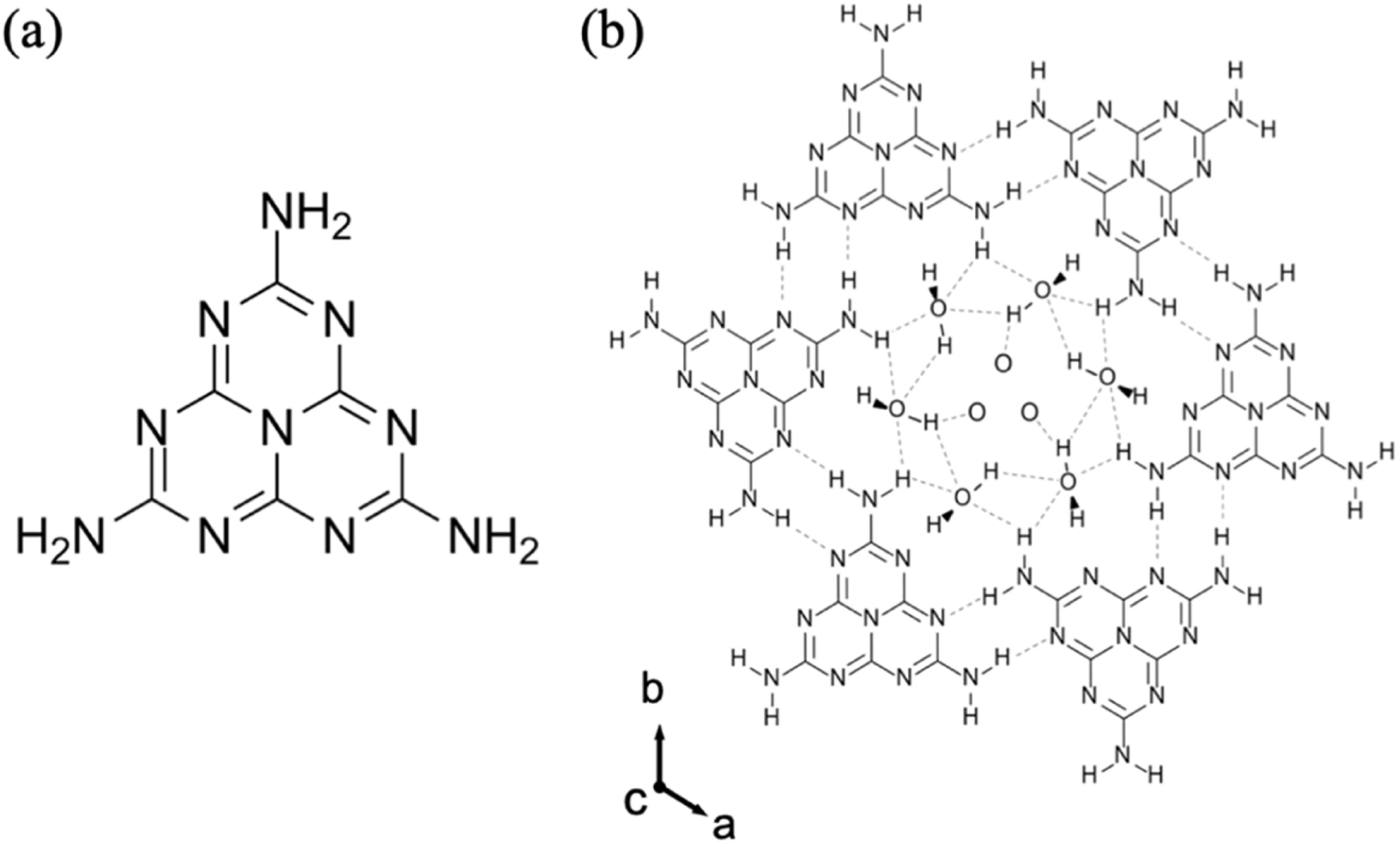
Molecular structures of (a) melem and (b) Mh; gray dotted lines represent intermolecular hydrogen bonds.

Melem (2,5,8-triamino-tri-s-triazine) (Figure 1a) as three amino groups (NH_2_) at its heptazine ring terminals and a crystal structure formed through hydrogen bonds and van der Waals interactions between the molecules. Melem possesses unique optical properties among CN materials.^5,6^ Melem exhibits photoluminescence (PL) in the near-UV (NUV) region, with a peak at λ_PL_ = 367.9 nm. The quantum yield of melem crystal, which indicates the luminescence efficiency, was as high as *Φ*_PL_ = 71%. A recent report suggests that one reason for the high quantum yield is delayed fluorescence due to TADF.^6,7^ According to Kiuchi et al., the fluorescence lifetime of melem is τ = 176 ns.^6^ In addition, it has been reported that NUV emission can be obtained from OLEDs using melem as the light-emitting layer.^8,9^ However, melon has a lower quantum yield than melem and shows no delayed fluorescence (*Φ*_PL_ = 7.4%, τ = 5.1 ns). This suggests that although melem is a melon building block, when it polymerizes in one dimension to form melon, it no longer exhibits TADF. Compared to materials that emit in the visible light region, there are few reports on TADF materials that exhibit NUV emission, such as melem.^7^

In the melem crystal, hydrogen bonds are formed between melem molecules via the nitrogen atoms of the heptazine ring and the hydrogen atoms of the amino group. Hydrogen bonds play a major role in the construction of crystal structure of melem. For this reason, melem molecules also form hydrogen bonds with water molecules and easily form hydrate crystals.

Melem hydrate (Mh) was reported by Makowski et al. in 2011.^10^ Mh can be produced from melem by sonication in water, vapor diffusion,^11^ stirring under reflux,^12^ and hydrothermal methods.^10^ The crystal structure of Mh has a helical, stacked structure of melem molecules in the *c*-axis direction, with a channel roughly 8.9 Å in diameter along the helix axis (Figure 1b). The Mh channels usually accommodate water molecules, but they are large enough to store gas and organic molecules, thus providing a high molecular adsorption capacity through dehydration. Mh has also been investigated as a precursor. Melon obtained through a hydrothermal polymerization process, using Mh as a precursor, exhibits superior photocatalytic hydrogen evolution efficiency and organic matter decomposition compared to conventional melon because of its improved crystallinity and increased surface area.^12,13^ The Mh luminescence properties are reported to be strongly affected by the amount of water it encapsulates. This indicates that the hydrogen bond between melem and water molecules within Mh has a significant impact on the luminescence mechanism of melem molecules.^6^ It is important to note that intentional dehydration of Mh results in emission at short wavelengths and high quantum yields not seen in other TADF molecules.^6^ Therefore, clarification of the Mh luminescence mechanism is an important requirement for the future molecular design of heptazine-based TADF molecules, especially those that actively take advantage of hydrogen bonds. In this study, we focused on parallelogram-shaped Mh (Mhp), a polymorph of melem hydrates recently reported by Dai et al.^11^ Mhp crystals have a parallelogram shape, Mh crystals a hexagonal prism shape. Mhp is a new polymorph, and its crystal structure and other basic physical properties have not yet been investigated in detail. The purpose of this study was to evaluate the crystal structure and other basic physical properties of Mhp and to elucidate the effect on the Mhp luminescence mechanism of hydrogen bonding between melem and hydrated water. Our study revealed that Mhp emits in the NUV region, similar to melem, and exhibits a high quantum yield and delayed fluorescence. We also found that the hydrogen bond between melem and hydration water had a significant effect on the Mhp emission mechanism and changed the emission wavelength.

## 2 Experimental Section

### 2.1 Materials

#### 2.1.1 Synthesis of Melem

1,3,5-Triazine-2,4,6-triamine (melamine) (5 g; purity: 99.0%; FUJIFILM Wako Pure Chem. Co., Ltd.; 139–00945) was placed in a tube furnace (KTF035N1; Koyo Thermo Systems Co., Ltd.) under an N_2_ atmosphere (purity: 99.99995%). The temperature of the material was increased at a rate of 1 °C min^–1^ from room temperature (approximately 20 °C) to 310 °C and maintained there for 5 h. The material was subsequently cooled to obtain melem. To remove unreacted melamine from the melem powder, *N*,*N*-dimethylformamide (DMF), a solvent in which melamine is soluble and melem is insoluble, was used. Melem powder (500 mg) was added to DMF (50 mL), sonicated for 15 min, and then centrifuged twice.

The obtained sample was confirmed to be melem by powder X-ray diffraction (XRD) and Fourier-transform infrared (FTIR) measurements (Figure S1 in the Supporting Information).

### 2.2 Vapor Diffusion Method

In this study, based on a previous study,^11^ we grew single crystals of Mhp using a mixture of ultradehydrated dimethyl sulfoxide (DMSO) and pure water as a good solvent and methanol as a poor solvent. Melem powder (45 mg) was added to DMSO (30 mL) and sonicated for 15 min.

The obtained solution (2 mL) was placed in a screw tube (6 mL), and pure water (200 μL) was added dropwise and sonicated for 15 min. The screw tube was then placed in a snap-cup (20 mL) containing methanol (4 mL) and sealed with a lid. The snap cup was then left at 30 °C in the dark for 7 days to grow crystals.

### 2.3 Ultrasonic Treatment

For the ultrasonic treatment, BRANSON 2510 Ultrasonic Cleaner was used and a solvent was prepared by mixing water and DMF. DMF is a poor solvent for melem, but it also has the property of being miscible with water. For this reason, it is easy to control the water content. The water content was varied from 5 to 40%, and the ultrasonic treatment was carried out using the following procedure. The water content is expressed as a percentage of the total volume of the mixed solution. First, a mixture of DMF and pure water (water content: 5–40%) was sonicated for 10 min. Then, 25 mL of the mixture was dispersed with melem (250 mg), and sonication and centrifugation were performed twice for 15 min. Furthermore, sonication and centrifugation were performed twice for 15 min with acetone. Finally, the obtained sample was left overnight and dried.

### 2.4 Solvothermal Method

In the solvothermal method,^14^ An AS ONE MMF-1 compact programmable electric furnace was used to heat the container. First, a mixture of DMF, pure water (50 mL; volume concentration of pure water: 15%), and melem (25 mg) was dispersed in a Teflon vessel and sonicated for 15 min. The Teflon container and lid were then tightly sealed with grease, and the container was sealed with a stainless-steel container. The stainless steel container was then heated to 180 °C at 2 °C min^–1^, held for 3 h, allowed to cool to 99 °C for 2 h, and then allowed to cool naturally.

### 2.5 Characterizations

XRD analyses were conducted using a diffractometer (Ultima IV, Rigaku) with a Cu Kα radiation source (λ = 1.5418 Å). Polarized light microscopy (POM) images were obtained and recorded under crossed-Nicol conditions using a microscope (SMZ1000, Nikon). Osmium-coated samples were used for scanning electron microscopy (SEM; FE-SEM SUPRA40; Carl Zeiss). The osmium coating was applied with a coater (Neoc-Pro; MEIWAFOSIS, Ltd.) using osmium (VIII) oxide (purity: 99.8%; FUJIFILM Wako Pure Chem. Co., Ltd.; 157–00404). The samples were attached to carbon tape. FTIR spectra for samples embedded in KBr pellets were acquired using a spectrophotometer (JASCO Corporation, FTIR-6100). In the KBr plate method for FTIR measurements used in this study, a sample was placed between two small pieces of KBr and the sample transmission spectrum obtained by pressure molding was measured. Because the pressurized crystals collapsed, a material-specific spectrum independent of the molecular orientation and optical interface was obtained. The optical anisotropy of the single-crystal sample was evaluated by measuring its FTIR spectrum using the reflection method with linearly polarized incident light. A VIRT-3000 (JASCO) instrument was used for these measurements. A gold vacuum-deposition film (thickness: 70 nm) was used as a reference sample for background correction. Thermogravimetric-differential thermal analysis (TG-DTA) was performed using a TG-DTA2010SA instrument (Bruker XS). TG-DTA curves were acquired in a dry N_2_ atmosphere at a heating rate of 5 °C min^–1^. An XtaLAB Synergy-S/NC diffractometer (Rigaku) was used for the single-crystal XRD measurements at room temperature (∼297 K). Mo Kα (λ = 0.71073 Å) was used as the light source and Olex2 software was used for single-crystal structure analysis.^15^ In the analysis, ShelXT was used to determine the initial phase and ShelXL was used to refine the structure.^16,17^ PL spectra and absolute PL quantum yields of the powdery samples were measured using a spectrometer (Quantaurus-QY, C11347–01; Hamamatsu Photonics, Ltd.) with the samples in quartz Petri dishes (A10095–03; Hamamatsu Photonics, Ltd.) PL measurements were performed in the emission wavelength range of 300–500 nm for all samples. A SHIMADZU RF-6000 was used to measure the temperature dependence of the PL spectra, and a CoolSpeK UV/CD USP-203 cryostat was used for cooling. Samples for the fluorescence lifetime measurements were prepared by sandwiching a powder sample between two quartz plates (20 × 20 mm^2^). Measurements were performed using a compact fluorescence lifetime measurement system (Quantaurus-Tau, C11367–01; Hamamatsu Photonics, Ltd.).

### 2.6 Calculation Methods

XRD profiles were calculated using the powder diffraction pattern package by employing the Visualization for Electronic and Structural Analysis (VESTA) program. Materials Studio (BIOVIA) was used as the calculation software, and energy level calculations were performed using the CASTEP plane wave basis set and general gradient approximation (GGA) with Perdew–Burke–Ernzerhof (PBE) as the correlation functional (Dassault Systemes BIOVIA).^18,19^ The details of the calculation are as follows: SCF tolerance threshold: 1 × 10^–6^ eV per atom; core treatment: all electrons were included in the calculation; basis set: DNP; basis file: 4.4.

The FTIR simulations were performed using Gaussian09 software (B3LYP/6–31G(d)).

## 3 Results and Discussion

### 3.1 Growth of Mhp Single Crystal

To determine the optimal method for growing Mhp single crystals, we first grew Mhp single crystals using vapor diffusion based on previous research.^11^ The precursor of Mhp, melem, was synthesized using melamine as the raw material.

As a result of evaluating the obtained samples using XRD, POM, and SEM, we conclude that we successfully obtained a single crystal of Mhp using the vapor diffusion method demonstrated in previous research.^11^ For detailed evaluation results of the obtained samples, see Figure S2.

Next, we discuss the growth of Mhp crystals using ultrasonic treatment. The melem was ultrasonically processed in a mixture of water and DMF.

**Figure 2:**
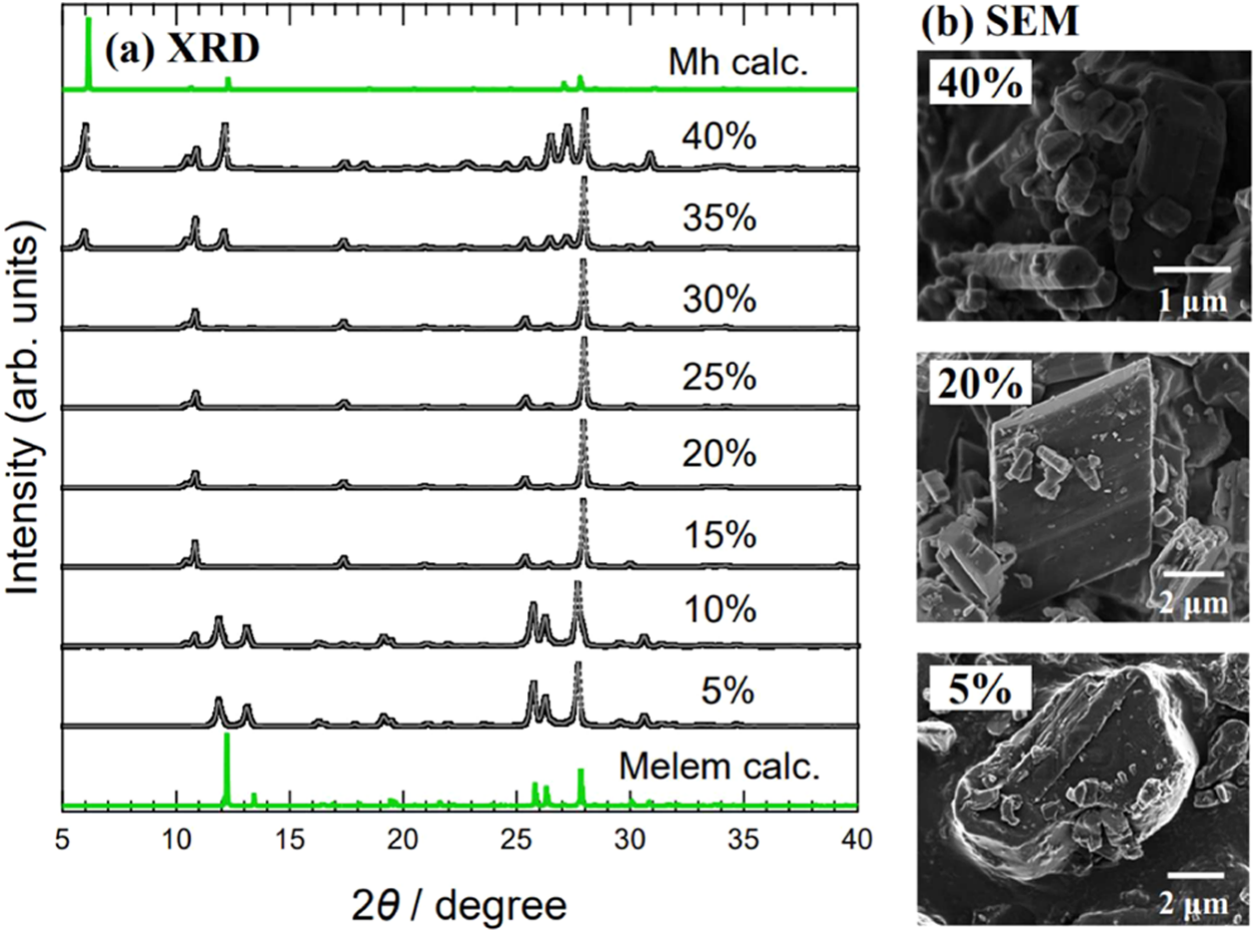
(a) XRD patterns of samples prepared by ultrasonic treatment. Samples were prepared by varying water content in ultrasonic treatment solution from 5 to 40%. Horizontal axis: diffraction angle 2θ; vertical axis: diffraction intensity. Green lines are simulated XRD patterns of melem crystal (Melem calc.) and Mh (Mh calc.). The simulations were performed using the crystal structures of melem crystal and Mh reported in previous studies.^10,20^ (b) SEM images of samples prepared with solutions containing 5, 20, and 40% water.

Figure 2a shows the powder XRD patterns of the obtained samples. The XRD patterns of each sample with varying solution water contents from 5 to 40% are displayed. As the water content increased, the XRD patterns changed. The sample with a water content of 5% matched the XRD pattern of melem crystals well. However, the sample with a 10% water content, in addition to the XRD pattern of melem crystals, contained the diffraction peaks characteristic of Mhp at 10.4 and 10.8°. In samples prepared with solutions containing 15–30% water, no diffraction peaks derived from melem crystals were observed, only the peaks characteristic of Mhp. In addition, for samples prepared using solutions with higher water contents of 35 and 40%, diffraction peaks characteristic of Mhp appeared, as well as diffraction peaks consistent with the XRD pattern of Mh.

Figure 2b shows a SEM image of a sample obtained by ultrasonic treatment. In the sample prepared with a 5% water solution, the Mhp and Mh crystals with clear facets cannot be seen. This is consistent with the sample XRD pattern, which was identified as a melem crystal. Next, for the sample prepared using a 20% water solution, parallelogram-shaped microcrystals of Mhp were observed, consistent with the XRD pattern of this sample, which showed only diffraction peaks characteristic of Mhp. However, the Mhp crystal size was only a few micrometers, approximately one-tenth the size of Mhp crystals grown using vapor diffusion. In the sample prepared using a 40% water-content solution, rod-shaped microcrystals were observed in addition to plate-shaped microcrystals. Mh is known to form rod-shaped single crystals,^11^ and this result is consistent with the XRD pattern, which showed the diffraction peaks of Mh.

From the above results, it was determined that when the proportion of water in the solution was increased during ultrasonic processing, the crystal structure of the major compound obtained gradually shifted from melem to Mhp to Mh. Samples prepared using 15–30% water-content solutions showed only the diffraction peaks of Mhp in the XRD pattern. Therefore, Mhp can be grown using ultrasonic treatment under these conditions. In this study, we used samples with a 20% water content to investigate the physical properties of ultrasonic-grown Mhp.

From the above discussion, we concluded that Mhp could be grown by sonication using a mixture of DMF and water. However, the size of the obtained crystals was only several micrometers, not large enough for structural analysis by single-crystal XRD measurements. Therefore, we focused on solvothermal growth, as single-crystal Mh samples several hundred μm in size have been grown by the hydrothermal method,^10^ a solvothermal method using water as the solvent.

**Figure 3:**
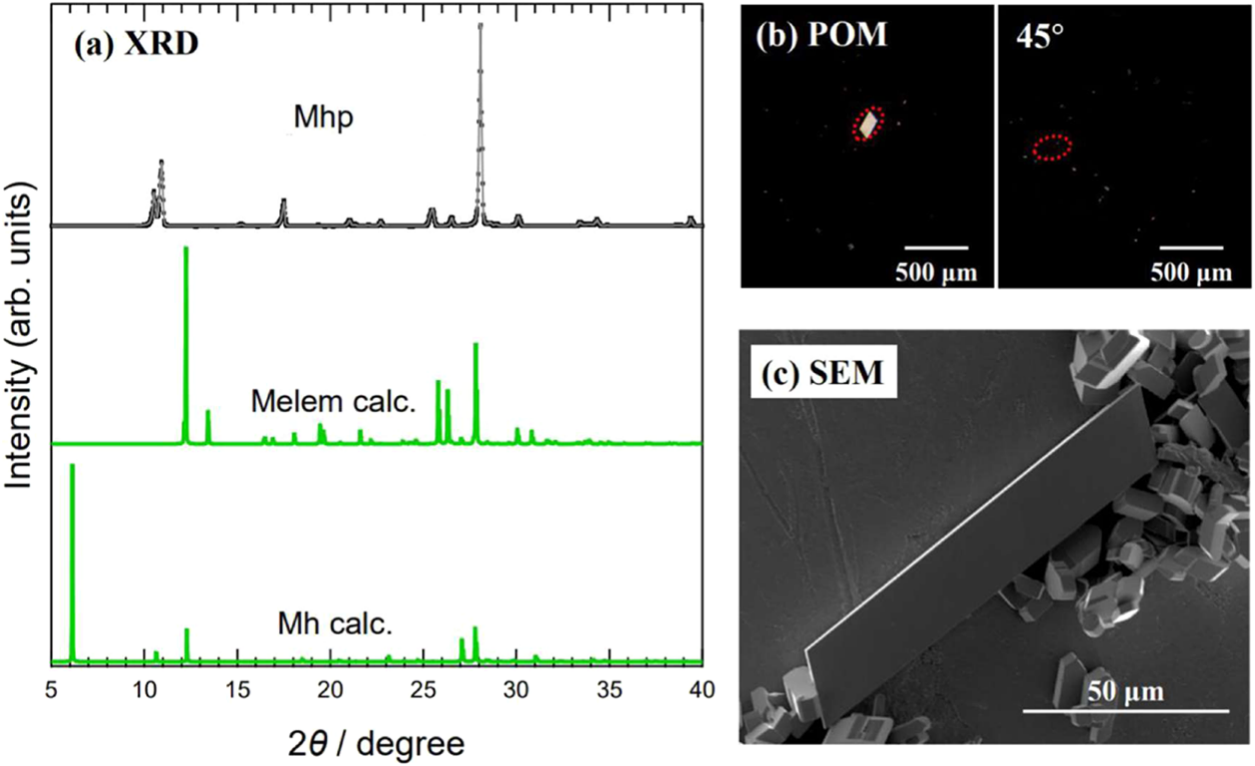
(a) XRD pattern of Mhp grown using solvothermal method; horizontal axis, diffraction angle 2θ; vertical axis, diffraction intensity. Green lines are simulated XRD patterns of melem crystal (Melem calc.) and Mh (Mh calc.). The simulations were performed using the crystal structures of melem crystal and Mh reported in previous studies.^10,20^ (b) POM image of Mhp sample. Image on right was obtained by rotating sample in image on left by 45°. (c) SEM image of Mhp sample.

Figure 3a shows the powder XRD measurements of the Mhp sample obtained via hydrothermal synthesis. The measured XRD pattern showed diffraction peaks at 2θ = 10.4 and 10.8°, which are characteristic of Mhp, unlike melem crystals and Mh. Figure 3b shows the sample POM image obtained under crossed-Nicols conditions. The transmitted light was detected under crossed-Nicols conditions, and complete extinction was observed by rotating the sample by 45°. Therefore, we concluded that the obtained Mhp sample is a single crystal. Figure 3c shows the SEM image of the obtained sample. The sample crystals have clear facets and are plate-like with a parallelogram shape, characteristic of Mhp. In addition, more plate-like crystals with greater thicknesses were observed than in Mhp grown using vapor diffusion. The Mhp crystals obtained by hydrothermal synthesis were larger than those grown by vapor diffusion or ultrasonic treatment, and crystals several hundred micrometers in size were observed. The SEM image shows many small crystals, but the XRD results show that they are all Mhp.

The results of FTIR and TG-DTA measurements of Mhp crystals grown using the vapor diffusion method, ultrasonic treatment, and solvothermal method (Figure S3) are in good agreement with the results of previous studies.^11^ There were no differences in crystal structure, chemical state, or thermal behavior depending on the growth method, and only the crystal size differed.

In the DTA results for Mhp, an endothermic reaction without a decrease in mass was observed at approximately 150 °C immediately after dehydration, and this endothermic reaction corresponds to a transition in the crystal structure. A detailed discussion of this structural transition is presented in Section 3.3 “Thermal Response of Mhp”.

### 3.2 Crystal Structure of Mhp

In this section, we describe the crystal-structure analysis of Mhp using single-crystal XRD measurements. In previous study,^11^ the powder XRD pattern of Mhp has been reported, but its crystal structure has not been reported.

**Figure 4:**
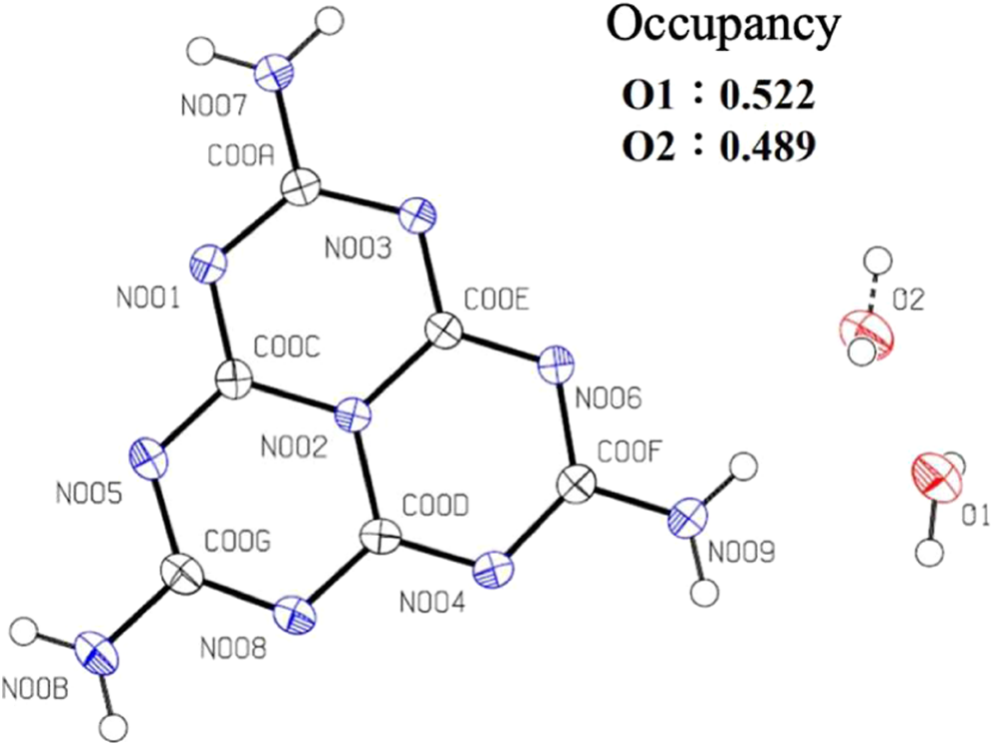
ORTEP drawing of Mhp single crystal. Black, blue and red ellipsoids represent carbon, nitrogen, and oxygen atoms, respectively. The ellipsoids are isosurfaces with a probability of 50% for the presence of atoms. The small white spheres are hydrogen atoms. The occupancy of the two oxygen atoms O1 and O2 was 0.522 and 0.489, respectively. PLATON software was used to create the drawing.^21^ The obtained atomic coordinates and occupancies are summarized in Table S1.

As shown in Figure S4, we measured the single-crystal XRD pattern of the Mhp single crystal obtained using the solvothermal method. By mapping the diffraction X-rays obtained onto the reciprocal lattice space, clear reciprocal lattice points were obtained. The Oak Ridge Thermal-Ellipsoid Plot Program (ORTEP) drawing obtained from the single-crystal X-ray structure analysis is shown in Figure 4. Ellipsoids in Figure 4 represent a 50% probability of atomic existence. The *R* factor of the obtained crystal structure was 4.15%. The molecular structure shown in Figure 4 included two adjacent water molecules, in addition to the melem molecule with its heptazine backbone. These results indicated that Mhp is a melem hydrate. The two water-molecule oxygen atoms in the ORTEP drawing were denoted O1 and O2, respectively. The interatomic distance between O1 and O2 was 2.04 Å, closer than the van der Waals radius, which indicates that the two adjacent water molecules cannot exist simultaneously. The occupancies of O1 and O2 were determined to be 0.522 and 0.489, respectively, as shown in the figure. Thus, a water molecule randomly located in one of two adjacent positions will have a spatial and temporal average probability of approximately 50% at each.^22^ The amount of water molecules encapsulated in the crystal can be calculated from the occupancy of water-molecule oxygen atoms.^10^ From the water occupancy obtained here, the amount of water molecules per mol of melem molecules in the Mhp single crystal was determined to be 1.01 mol, consistent with the estimate from the TG-DTA results of 1.00 mol of water per mol of melem molecules in the Mhp single crystal.

The results of the single-crystal structure analysis of Mhp confirmed that Mhp is a melem hydrate containing a disordered amount of hydration water molecules.

**Figure 5:**
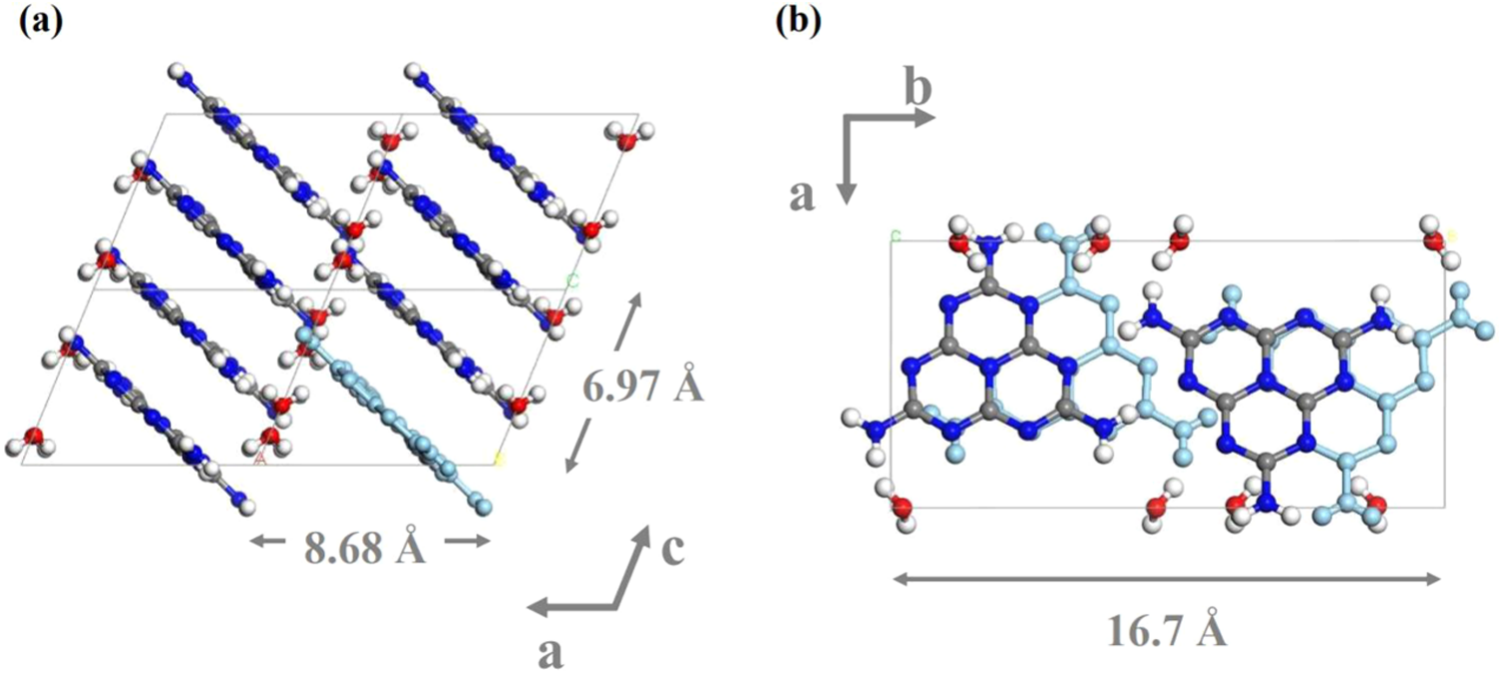
Crystal structure of Mhp single crystal obtained by single-crystal structure analysis: (a) view from the *b*-axis direction; (b) view from the *c*-axis direction. Gray, blue, red, and white spheres represent carbon, nitrogen, oxygen, and hydrogen atoms, respectively. Some melem molecules are shown in light blue to make the crystal structure easier to understand.

The Mhp crystal structure obtained from the crystal structure analysis is shown in Figure 5. Figure 5a,b show the crystal structure viewed from the *b*- and *c*-axis directions, respectively. As shown in Figure 5a, the melem molecules are stacked in layers oriented perpendicular to the ac-plane, because the parallelogram plane of the Mhp single crystal corresponds to the ac-plane. This figure shows the molecular arrangement when looking down at the parallelogram plane. In addition, Figure 5b shows that the melem molecules are aligned in the same direction along the *a*-axis and rotated 60° along the *b*-axis, whereas they are stacked alternately in the same direction along the *c*-axis. Water molecules are distributed around the amino group (NH_2_) of melem.

From the above discussion, it is clear that in the Mhp structure, melem molecules are stacked in layers and water molecules are localized around the amino groups of the melem molecules. The crystal structure of Mhp is very different from that of Mhr. Mhp has a structure in which the molecular layers of melem are stacked in the *c*-axis direction, but Mhr has the arrangement of melem molecules in helical structures along the *c*-axis.^11^

Information obtained from the crystal structure analysis is presented in Table 1.

**Table 1:** Lattice Constants and Other Crystal Structure Information Obtained through Crystal Structure Analysis

		space group	*a*/Å	*b*/Å	*c*/Å	α/°	β/°	γ/°	refs
Mhp	monoclinic	*P*2_1_/*c*	8.6846(4)	16.6680(5)	6.9674(3)	90	112.487(5)	90	this work
melem	monoclinic	*P*2_1_/*c*	7.3992(1)	8.6528(3)	13.3816(4)	90	99.912(2)	90	(20)
Mh	trigonal	*R*3̅*c*	28.790(4)	28.790(4)	6.6401(13)	90	90	120	(10)
	monoclinic	*P*21/*c*	8.6040(17)	16.630(3)	6.8840(14)	90	111.91(3)	90	(23)

The lattice constants and other information obtained from Mhp crystal structure analysis are listed in Table 1, top row. The crystal structure information on previously reported crystals with melem molecules as building blocks is shown for comparison. The lattice constants of the melem crystals in the second row and the Mh crystals in the third row differ from those of Mhp. In contrast, the fourth row shows the crystal structure of the material reported as melem hydrate in 2022, which has a different lattice constant than that of Mh.^23^ Comparison of the lattice constants and molecular arrangements with those of Mhp indicates that the crystal structures of this melem hydrate and Mhp are identical. Therefore, this study was not the first to identify the Mhp crystal structure. The previous study reported a crystal structure similar to that of Mhp and discussed the synthesis of poly(triazine imide) (PTI), a two-dimensional CN polymer synthesized by heating the eutectic salts of Dicyandiamide (DCDA) with alkali metals. When the anion in the alkali metal eutectic was changed, PTI was not synthesized using certain anions; melem hydrate was formed instead.

The melem hydrate obtained showed plate-like and crystal-like Mhp. The structure of this platelet crystal, obtained by structural analysis using single-crystal XRD measurements, was consistent with the Mhp structure obtained in this study. However, the XRD pattern of powder containing this melem hydrate showed broad diffraction peaks alongside the sharp diffraction peaks of melem hydrate. The powder was brown in color, suggesting that it might contain degraded melem.^24^ In addition, the basic physical properties of this melem hydrate, such as thermal response and optical properties, were not discussed in detail. In light of the above, we believe that the Mhp grown in this study has advantages in terms of crystallinity and purity, and that it is meaningful to discuss the thermal response and optical properties of Mhp.

### 3.3 Thermal Response of Mhp

**Figure 6:**
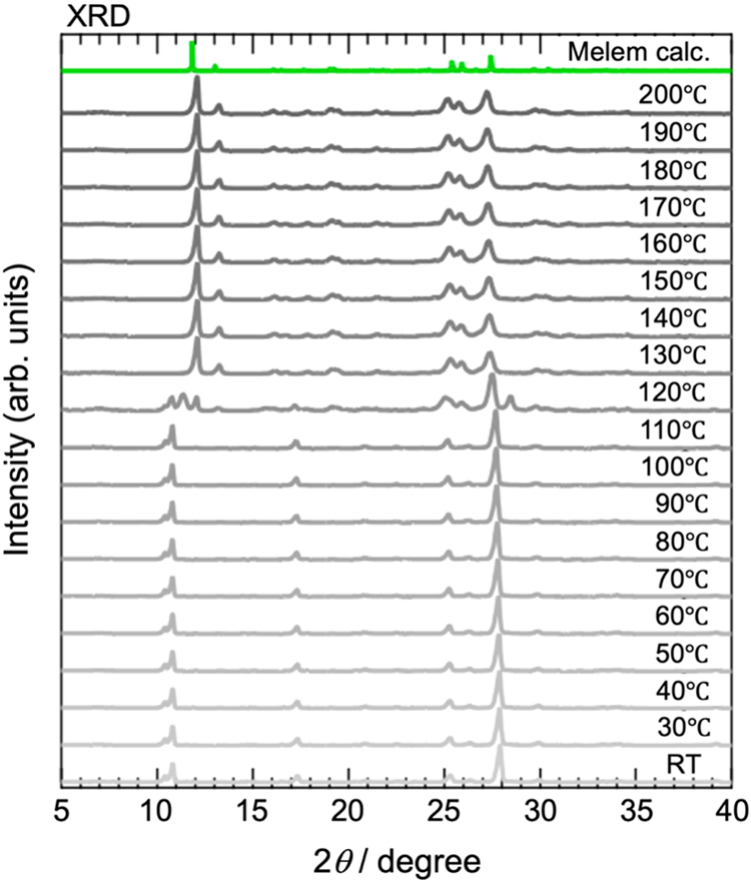
Results of temperature-variable powder XRD measurement: Horizontal axis, diffraction angle 2θ; vertical axis, diffraction intensity. XRD measurements were taken at 10 °C intervals as temperature was increased from room temperature to 200 °C at a rate of 5 °C min^–1^. Note that the increase was paused and the temperature held constant during the XRD measurements. Green line is simulated XRD pattern of melem crystal (Melem calc.). The simulation was performed using the crystal structure of melem crystal and Mh reported in previous studies.^20^ The simulated XRD pattern has been shifted 0.4° to the lower angle side to take into account the deviation in the diffraction angle due to height adjustment of sample stage.

As discussed above, the TG-DTA measurements of Mhp (Figure S3) indicated an endothermic reaction accompanied by a mass decrease at roughly 120 °C and an exothermic reaction without a mass decrease at roughly 150 °C. These correspond to dehydration at roughly 120 °C and a transition in the crystal structure at roughly 150 °C. In previous research, heating Mhp at 200 °C for 3 h reportedly caused the crystal structure to shift to that of melem.^11^ Therefore, we performed temperature-variable powder XRD measurements to investigate the crystal-structure changes in more detail. The results of XRD measurements taken at 10 °C intervals while heating from room temperature to 200 °C at 5 °C min^–1^ are shown in Figure 6.

We will discuss the changes in the XRD pattern in three temperature regions: (1) from room temperature to 110 °C; (2) from 120 to 130 °C; and (3) from 130 to 200 °C.

(1) From room temperature to 110 °C, no significant change was observed in the XRD pattern, indicating that the Mhp crystal structure hardly changed in this temperature range. This is consistent with the results of the TG-DTA measurements, in which almost no dehydration was observed at room temperature.
(2) At 120 °C, a change in the XRD pattern was observed. This was approximately the same temperature as that of the dehydration observed in the TG-DTA measurements. Because of the dehydration, the crystal structure of Mhp began to alter. However, in the XRD pattern at 120 °C, diffraction peaks were observed that were neither Mhp nor melem crystals. This suggested the existence of an intermediate state between dehydration and the transition to melem crystals. In the TG-DTA measurement, there was also a difference of roughly 10 °C between the end of the mass decrease accompanying the dehydration and the exothermic reaction. Therefore, it is possible that an intermediate state existed in this region.
(3) At temperatures above 130 °C, the XRD pattern matched that of melem crystals. Therefore, Mhp had already undergone a structural transition to melem crystals in this temperature range. Note that the temperature at which Mhp began to display the melem crystal structure (130 °C) was 20 °C lower than the temperature at which exothermic reactions were observed in the TG-DTA (150 °C). This was due to the effect of the heating holding time during the XRD measurements. The heating rates for both the temperature-variable XRD measurement and the TG-DTA measurement were 5 °C min^–1^. However, in the temperature-variable XRD measurements, the heating was paused and the temperature held constant during the measurements themselves. Therefore, it is possible that the crystal structure transition occurred at a lower temperature than that observed in TG-DTA. Based on the above discussion, we concluded that the structural transition from Mhp to melem crystals was triggered by dehydration.

**Figure 7:**
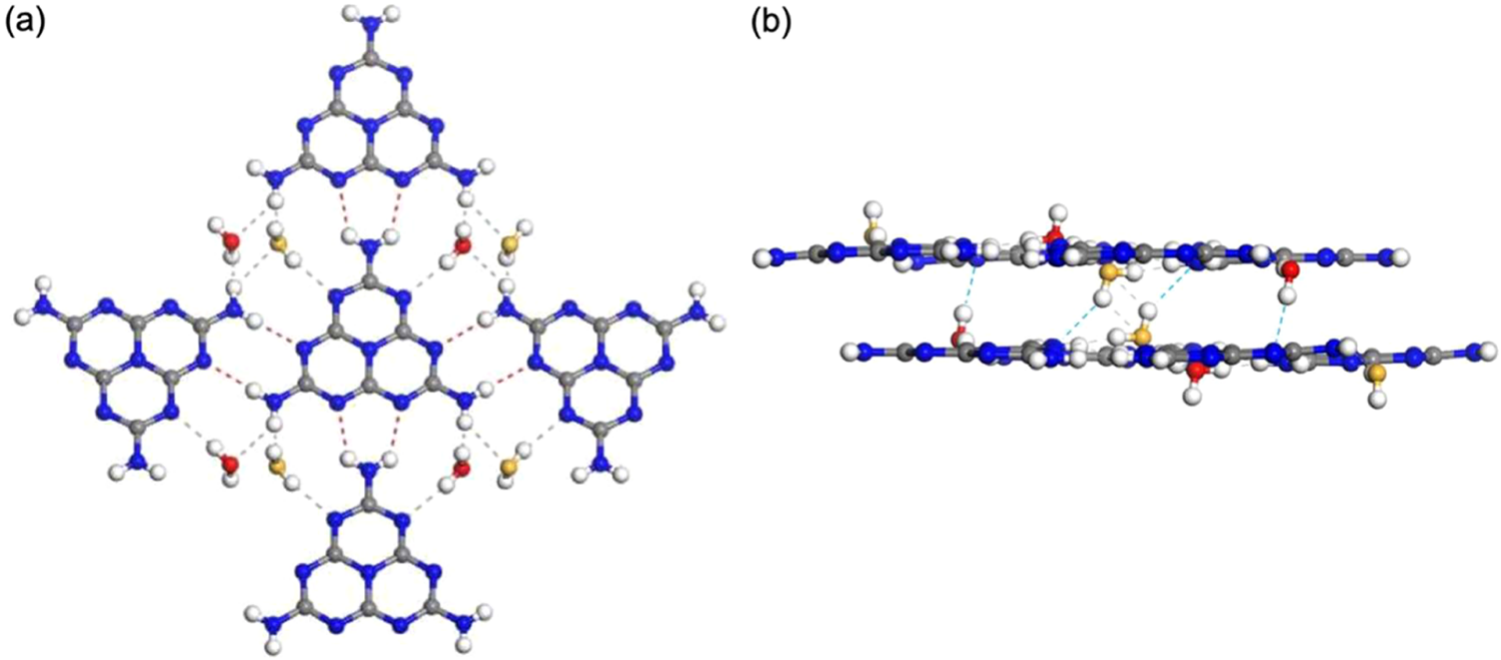
Hydrogen bonds between molecules in Mhp, based on crystal structure model obtained from comparison with XRD results: (a) hydrogen-bond framework of intralayer crystal structure (ab-plane); (b) hydrogen-bond framework viewed along direction of molecular layer stacking. Dotted lines indicate hydrogen bonds. Water molecule disorder is distinguished by oxygen atom color (red or yellow).

The crystal structure transition upon dehydration occurs because the loss of water molecules disrupts the hydrogen-bond framework. To consider the hydrogen bonds that support the Mhp crystal structure, the hydrogen bonds between melem molecules and between melem and water molecules are shown in Figure 7.

Figure 7a shows the hydrogen bonds within the ab-plane of the Mhp crystal structure. Melem molecules formed hydrogen bonds between terminal NH_2_ group hydrogens and heptazine-skeleton nitrogen atoms (red dotted lines in the figure). Hydrogen bonds between melem molecules within the ab-plane can be classified into two types: “side-to-side,” which formed bonds in the horizontal direction as shown in the figure, and “head-to-tail,” which formed bonds in the vertical. The average hydrogen-bond distances between melem molecules were roughly 2.12 Å for the side-to-side bonds and 2.43 Å for the head-to-tail. Therefore, the side-to-side bonds that formed were stronger hydrogen bonds than the head-to-tail, because their bond distance was approximately 0.3 Å shorter. In contrast, Figure 7a shows that hydrogen bonds formed between water and melem molecules (represented by gray dotted lines). The disordered water molecules are distinguished by red or yellow oxygen-atom colors. Hydrogen bonds between water and melem molecules formed between hydrogens of melem molecule NH_2_ groups and oxygens of water molecules, as well as between hydrogens of water-molecule OH groups and the nitrogen of melem molecule heptazine backbones. The average hydrogen bond length between a water and a melem molecule was 2.15 Å. These hydrogen bonds indirectly connected melem molecules in the ab-plane.

Figure 7b shows the hydrogen bonds between molecular layers. Unlike the behavior in the ab-plane, no hydrogen bonds formed between melem molecules. In contrast, hydrogen bonds formed between hydrogens of water molecule OH groups and nitrogen of the melem molecule heptazine backbone (blue dotted lines). The average distance between these hydrogen bonds was 2.18 Å. These hydrogen bonds indirectly connected melem molecules in the interlayer direction. From the above discussion, we found that hydrogen bonds between melem molecules formed two-dimensionally in the ab-plane, and hydrogen bonds involving water molecules formed both in the ab-plane and between molecular layers. The dehydration-induced structural transition of Mhp was likely caused by the elimination of these water molecule-related hydrogen bonds. In particular, hydrogen bonds involving water molecules between the molecular layers contributed to the stabilization of the layered structure.^25^ Melam, an intermediate between melamine and melem, is reported to also form layered hydrates such as Mhp. Melam crystals also rearrange their molecules upon dehydration, and the layered structure is not maintained.^26^ Thus, the data suggest that water molecules played an important role in the stabilizing of the Mhp melem-molecule layered structure.

### 3.4 Optical Anisotropy of Mhp Single Crystals

As shown in Figure 5, single-crystal structure analysis revealed that the melem molecules in Mhp were arranged perpendicular to the parallelogram crystal plane (ac-plane), suggesting that Mhp single crystals exhibited optical anisotropy. Therefore, we performed FTIR measurements using direct reflection to evaluate the optical anisotropy of Mhp single crystals.

Evaluating the optical anisotropy required distinguishing between the anisotropies of melem molecules and water molecules. However, in the FTIR spectrum, the stretching vibrations of the melem NH groups and those of the water OH groups (in light water) were observed to overlap in the 3000–3500 cm^–1^ region. Therefore, we grew a single-crystal sample of Mdp, a heavy-water hydrate (D_2_O), and investigated its optical anisotropy. Deuterium (D) has twice the mass of light hydrogen because it has one more neutron. Therefore, the wavenumber of the IR absorbed by heavy water is roughly 1/√2̅ that of light water, and the OD stretching-vibration absorption appeared at 2100–2600 cm^–1^.^27^ Mdp single crystals were grown by the Mhp solvothermal procedure, simply changing light for heavy water. To confirm that the Mdp single crystals had grown properly, XRD and TG-DTA were performed and compared with the results for Mhp obtained by the solvothermal method (Figure S5). The XRD patterns were consistent, indicating that Mhp and Mdp had the same crystal structures. Furthermore, the results of the TG-DTA measurements of Mhp and Mdp showed both endothermic and exothermic reactions at the same temperatures, indicating that the hydration-water thermal behavior is the same in both.

**Figure 8:**
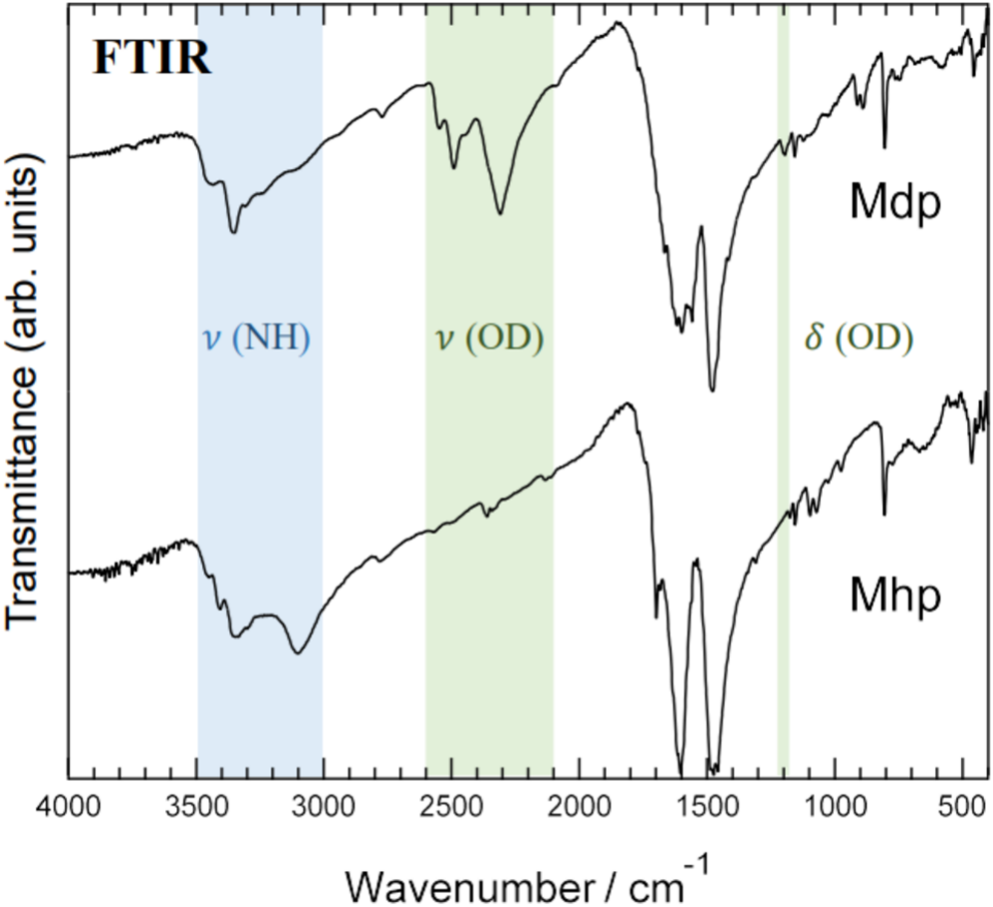
Comparison of FTIR spectra of Mhp and Mdp grown by the solvothermal method. Horizontal axis, wavenumber; vertical axis, transmittance.

Figure 8 compares the FTIR spectra of Mhp and Mdp. Mdp showed new absorption peaks, not observed for Mhp, near the 2100–2600 and 1200 cm^–1^ wavenumber regions; the former corresponds to the region where the OD-group stretching vibration appears and the latter to the region where the OD-group angular vibration peak appears. The absorption peak observed in the wavenumber region 3000–3500 cm^–1^ in Mdp had a shape different from that of Mhp. This is because, in Mdp, the contribution of the light water OH-group stretching vibration was reduced because heavy water had replaced the Mhp light water. Only a peak originating from the stretching vibration of the melem molecule amino group was observed.

**Figure 9:**
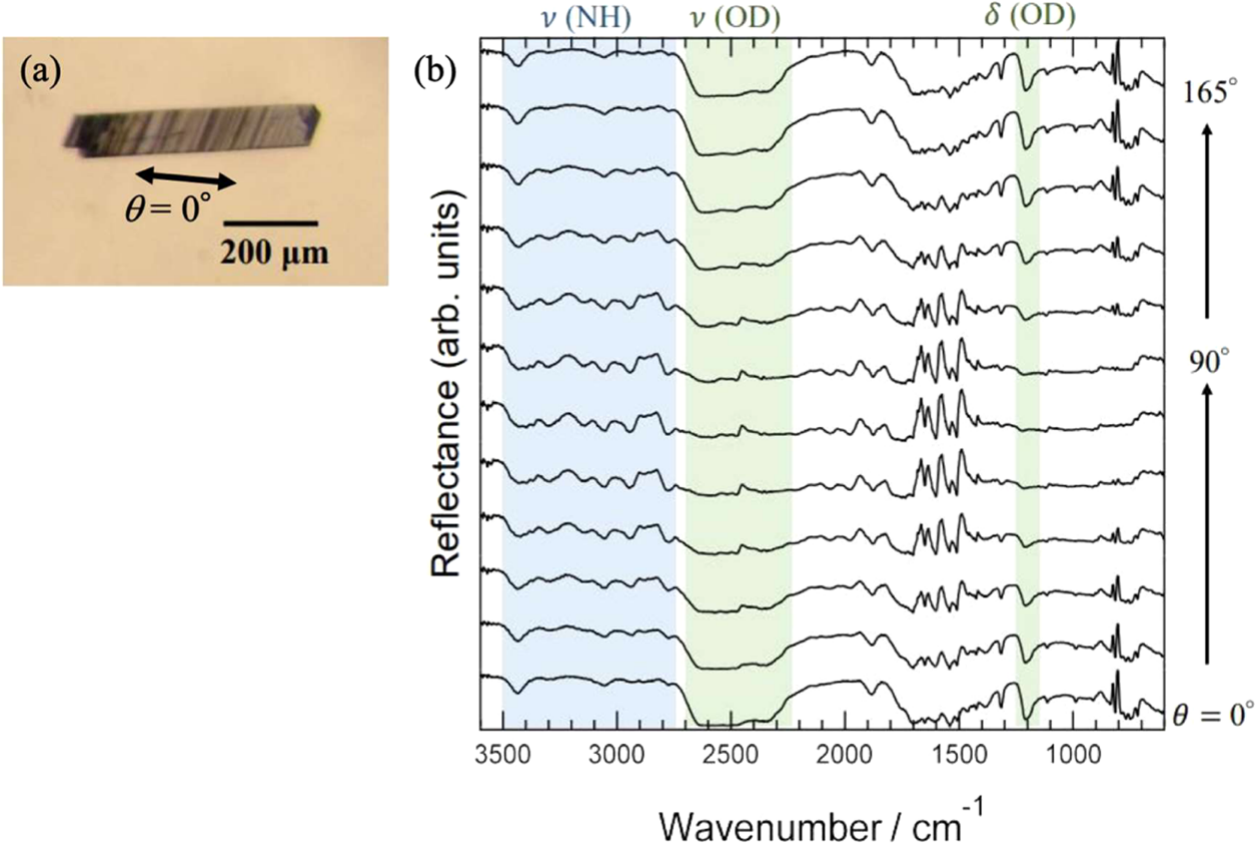
FTIR spectra of Mdp single crystal measured by direct reflection method: (a) photograph of single-crystal sample used for measurement and polarization direction of incident IR light; (b) reflection spectrum of Mdp single crystal. Horizontal axis is wavenumber, vertical axis reflectance. Polarization direction of incident light θ was rotated in steps of 15° from 0 to 165°, and spectra measured at each angle are shown from bottom to top. ν and δ represent stretching and bending vibrations, respectively.

Next, optical anisotropy was investigated by performing FTIR measurements on Mdp single crystals using the direct-reflection method. Figure 9a shows a photograph of the single-crystal sample used for the measurement. The arrows in the figure indicate the direction of polarization at θ = 0°; the direction of incidence is perpendicular to the surface of the parallelogram crystal. A sample with a long side of about 400 μm was used for the measurements. Figure 9b shows the FTIR spectra of the Mdp single crystal measured at each θ angle as θ was increased in steps of 15° from 0 to 165°. It can be seen that the spectra contain a polarization direction dependence. In fact, two types of polarization direction dependence are observed. The first appears in the wavenumber regions near 1200 and 2200–2700 cm^–1^. The absorption peak intensities in this region are at their maximum near θ = 150° and their minimum near θ = 60°. These wavenumber regions correspond to absorption due to the vibration of the heavy-water OD group, and the observed θ-dependence indicates the anisotropy of the heavy-water molecule orientation.

The second region of polarization dependence is that near 2800–3500 cm^–1^. The absorption peaks in this region reach their maximum intensity at roughly θ = 75° and their minimum at roughly θ = 165°. In these regions, absorption appears due to the vibration of the melem molecule NH_2_ group, and the observed dependence indicates the melem-molecule orientation anisotropy.

**Figure 10:**
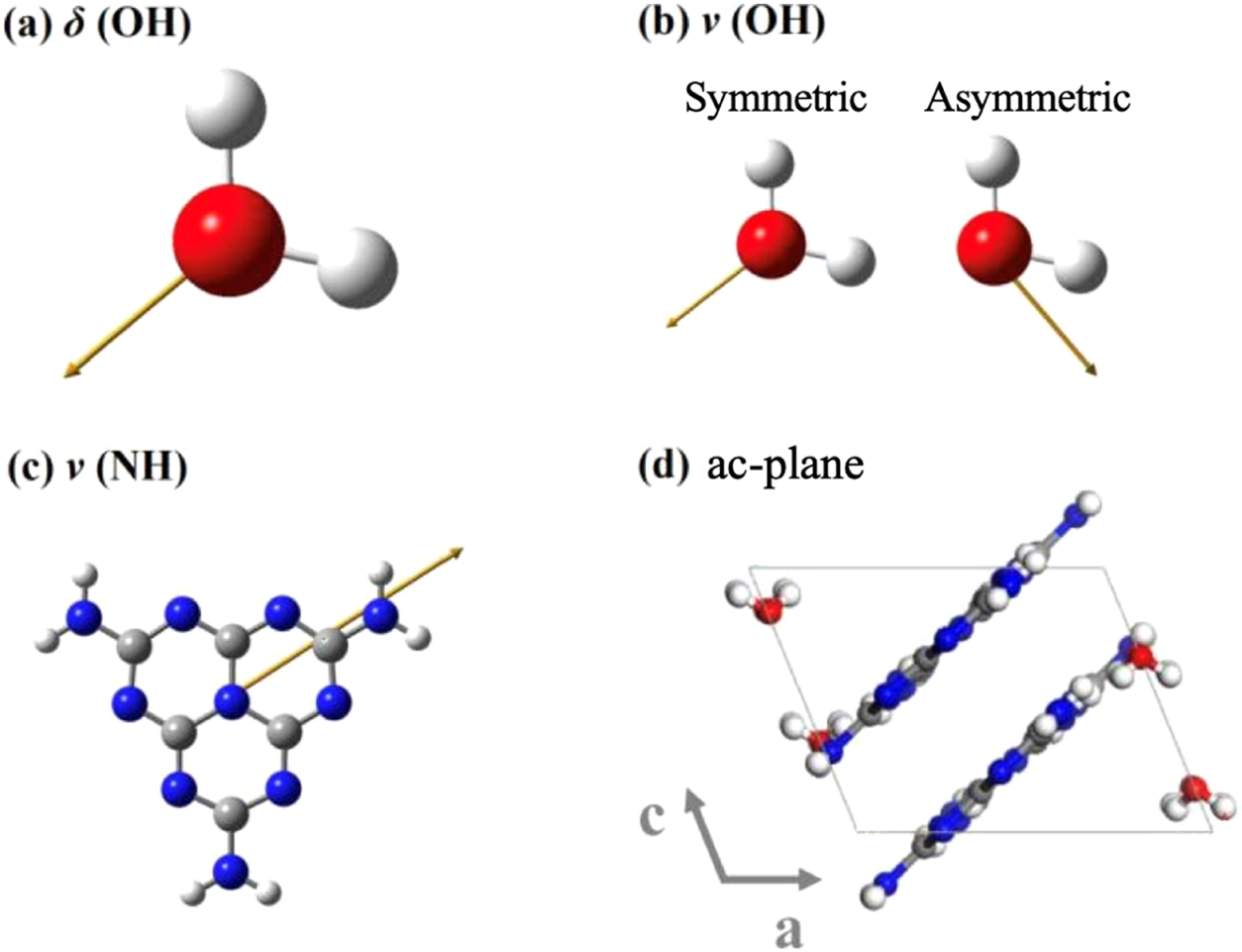
Electric dipoles generated by molecular vibrations resulting from IR absorption, simulated through density functional theory (DFT) calculations. Yellow arrows indicate dipole direction. ν and δ represent stretching and bending vibrations, respectively. (a) Bending vibration of light-water OH group; (b) stretching vibrations of light-water OH group; (c) stretching vibration of melem-molecule NH_2_ groups; (d) molecular arrangement in the ac-plane of Mhp.

We now discuss the reasons for the different polarization direction dependencies exhibited by heavy water and melem molecules in the direct reflection spectra. IR absorption intensity is generally maximized when the electric dipole induced by molecular vibration parallels the polarization direction of the incident light and is minimized when the dipole is perpendicular. Figure 10 shows the simulated dipole induced by IR absorption for the vibrations for which polarization direction dependence was observed.

Figure 10a shows the bending vibration dipole of the light-water OH group, 12(b) shows the stretching vibration dipoles. Because the dipole directions do not change between light and heavy water, the calculation results for light water are used in this discussion. Because symmetric and asymmetric stretching vibrations exist, two types of dipole orientations exist in the OH-group stretching vibration. Among the dipoles induced by OH group vibrations, those with bending and symmetric stretching vibrations are parallel, whereas those with asymmetric stretching vibrations are oriented differently. In the FTIR spectra shown in Figure 9, the OD-group bending and stretching vibrations exhibit a similar polarization direction dependence, suggesting that the symmetric stretching vibration of the OD group is strongly observed in the heavy water of Mdp. Figure 10c shows the dipole induced by the stretching vibrations of the melem molecule NH_2_ groups. These dipoles are parallel to the melem molecular plane. Based on the above discussion, we considered the dipole orientation in the ac-plane. Figure 10d shows the Mhp molecular arrangement in the ac-plane. The dipoles of all the molecules lie approximately in the ac-plane, although they contain water disorders. The melem molecules are stacked in layers, and the dipoles generated by the stretching vibrations of the two molecules are oriented in the same direction. Therefore, water and melem molecules should exhibit different polarization direction dependences. This explains why the absorption due to the OD vibration of heavy water and the vibration of the melem amino group showed different polarization direction dependencies in the Mdp FTIR spectra, as shown in Figure 9. These results are explained based on the crystal structure of Mhp obtained from the single-crystal structure analysis shown in Figures 4 and 5; they support this crystal structure.

### 3.5 Optical Properties of Mhp

In this section, we focus on the optical properties of Mhp. As mentioned in the Introduction, melem is a TADF material with a high quantum yield and emission in the near-ultraviolet region. Mhp is built of melem building blocks, and its optical properties are expected to be similar. In addition, new optical properties of Mhp are expected to be observed because of the hydrogen-bond framework, which differs from that of melem crystals, and the effects of hydration water.^28,29^ Therefore, we investigated the optical properties of Mhp and discuss them in terms of its crystal structure and hydration water.

**Figure 11:**
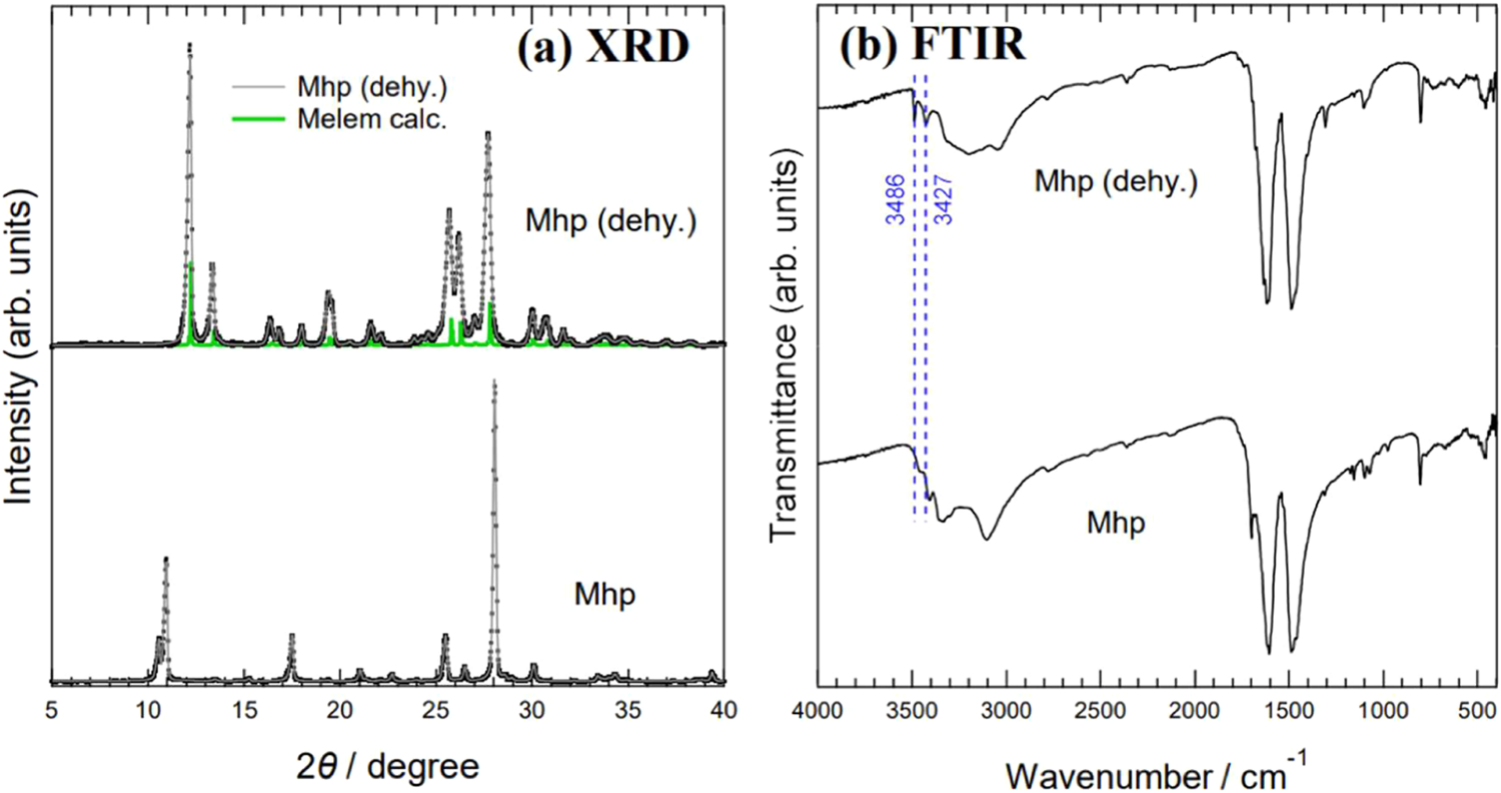
(a) XRD results of Mhp (dehy.) prepared by heating at 200 °C for 3 h. Horizontal axis is diffraction angle 2θ, vertical axis diffraction intensity. Green line represents simulated XRD pattern of melem crystal (Melem calc.). The simulation was performed using the crystal structure of melem crystal and Mh reported in previous studies.^20^ (b) FTIR spectrum of Mhp (dehy.) Horizontal axis is wavenumber, vertical axis transmittance.

To compare the optical properties of Mhp, we prepared Mhp samples that were heated and dehydrated. Based on the temperature dependence of the XRD results shown in Figure 6, Mhp was heated at 200 °C for 3 h to prepare a sample whose structure had been transferred to the melem crystal by dehydration. The sample obtained using this procedure was denoted Mhp (dehy.) XRD and FTIR spectra of Mhp (dehy.) are shown in Figure 11. Figure 11a shows the powder XRD patterns of Mhp and Mhp (dehy.) The XRD pattern of Mhp (dehy.) is almost identical to that of melem. This is because the Mhp was heated to a higher temperature than that at which Mhp undergoes structural transition to melem. Figure 11b shows the FTIR spectra of Mhp and Mhp (dehy.), in which two peaks characteristic of melem were observed at 3427 and 3486 cm^–1^.^20^ These results indicate that Mhp (dehy.) is a sample in which Mhp underwent a structural transition to melem by dehydration.

**Figure 12:**
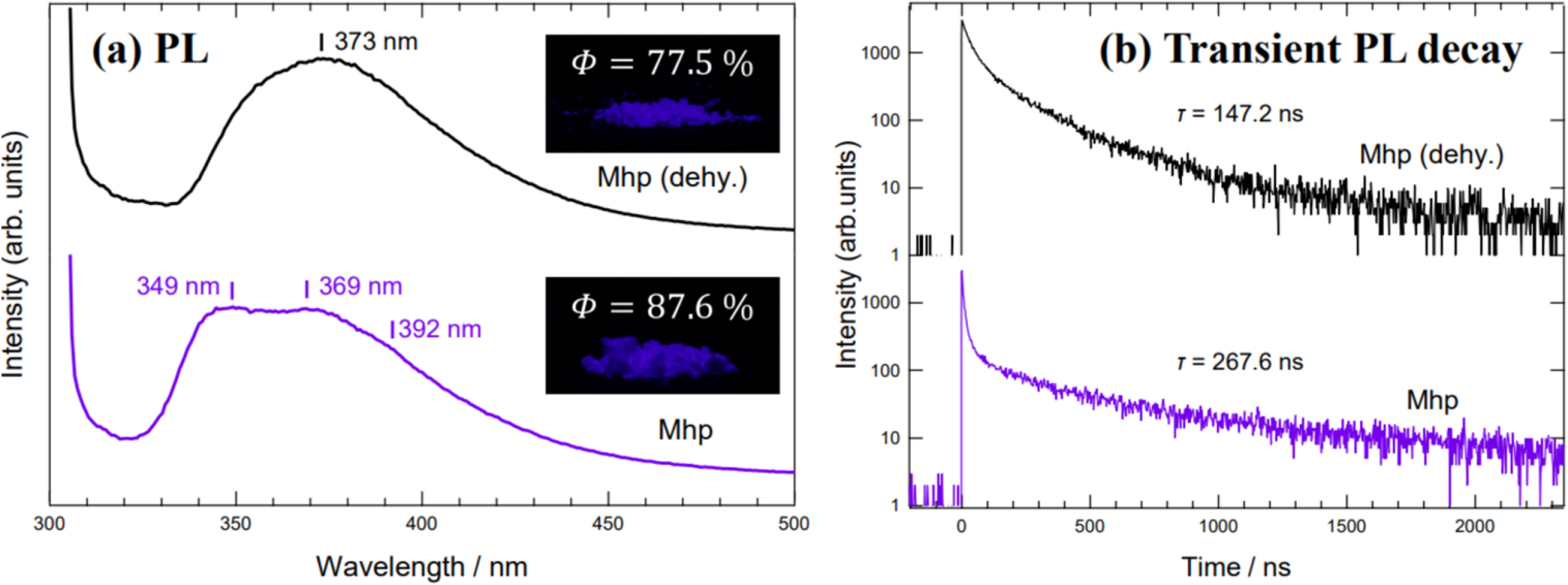
Comparison of optical properties of Mhp and Mhp (dehy.) All measurements were performed at room temperature. (a) PL spectra of Mhp and Mhp (dehy.); horizontal axis is emission wavelength, vertical axis luminescence intensity. Excitation light wavelength: 300 nm. Therefore, the spectrum intensity near 300 nm is caused by the excitation light. The photographs show the luminescence of each sample. Insets show the obtained quantum efficiency (*Φ*) values. (b) Fluorescence lifetime measurement results for Mhp and Mhp (dehy.); excitation light wavelength is 340 nm, emission wavelength 370 nm. Horizontal axis shows the time from the start of emission, vertical axis the logarithm of the luminescence intensity. τ represents the fluorescence lifetime obtained by fitting analysis of the PL decay curves.

Figure 12a presents PL spectra of Mhp and Mhp (dehy.) and photographs of the PL emission of each sample. Both samples exhibited violet emission in the near-ultraviolet region at 350–400 nm. The emission peak wavelength of Mhp (dehy.) is 373 nm, nearly identical to that of the reported emission peak of melem crystal (370 nm).^6^ In contrast, Mhp emits light over a wider wavelength range, with peaks at 349 and 369 nm and a peak shoulder at roughly 392 nm. Both samples exhibited high quantum yields, 87.6% for Mhp and 77.5% for Mhp (dehy.). Figure 12b shows the results of the fluorescence lifetime measurements for Mhp and Mhp (dehy.). In both cases, the luminescence intensity decayed within a few hundred nanoseconds. Fluorescent materials typically decay in the range of tens of nanoseconds, so Mhp and Mhp (dehy.) exhibited long fluorescence lifetimes.

In Figure 12b, the slope of the graph also changes, showing both prompt fluorescence with a short lifetime and delayed fluorescence with a long lifetime. Therefore, assuming that the lifetime is composed of two components, we performed a fitting analysis of the fluorescence lifetime graph in Figure 12b. The analysis results are shown in Table 2. The detailed analysis method is described in the Supporting Information.^30^ As shown in Table 2, Mhp and Mhp (dehy.) exhibited prompt fluorescence for tens of nanoseconds and delayed fluorescence for hundreds of nanoseconds. Compared to the previously reported melem, Mhp (dehy.) showed similar fluorescence lifetime for both prompt and delayed components, consistent with having undergone a structural transition to melem. In contrast, Mhp exhibits delayed fluorescence with a longer lifetime than Mhp (dehy.) or melem.

**Table 2:** Parameters Obtained from Fitting Analysis of Fluorescence Decay Curve for Mhp and Mhp (dehy.) (Figure 12b)^a^

	prompt		delayed		
	*A*_1_	τ_1_/ns	*A*_2_	τ_2_/ns	
Mhp	2475	22.9	234	447	this work
Mhp (dehy.)	2379	39.3	551	228	this work
melem	2793	31.5	803	266	(6)

a *A*_1_ and *A*_2_ are the intensities of prompt and delayed fluorescence at *t* = 0. τ_1_ and τ_2_ are the fluorescence lifetimes of the prompt and delayed fluorescence. For comparison, the Melem parameters reported in a previous study are shown.^6^

**Figure 13:**
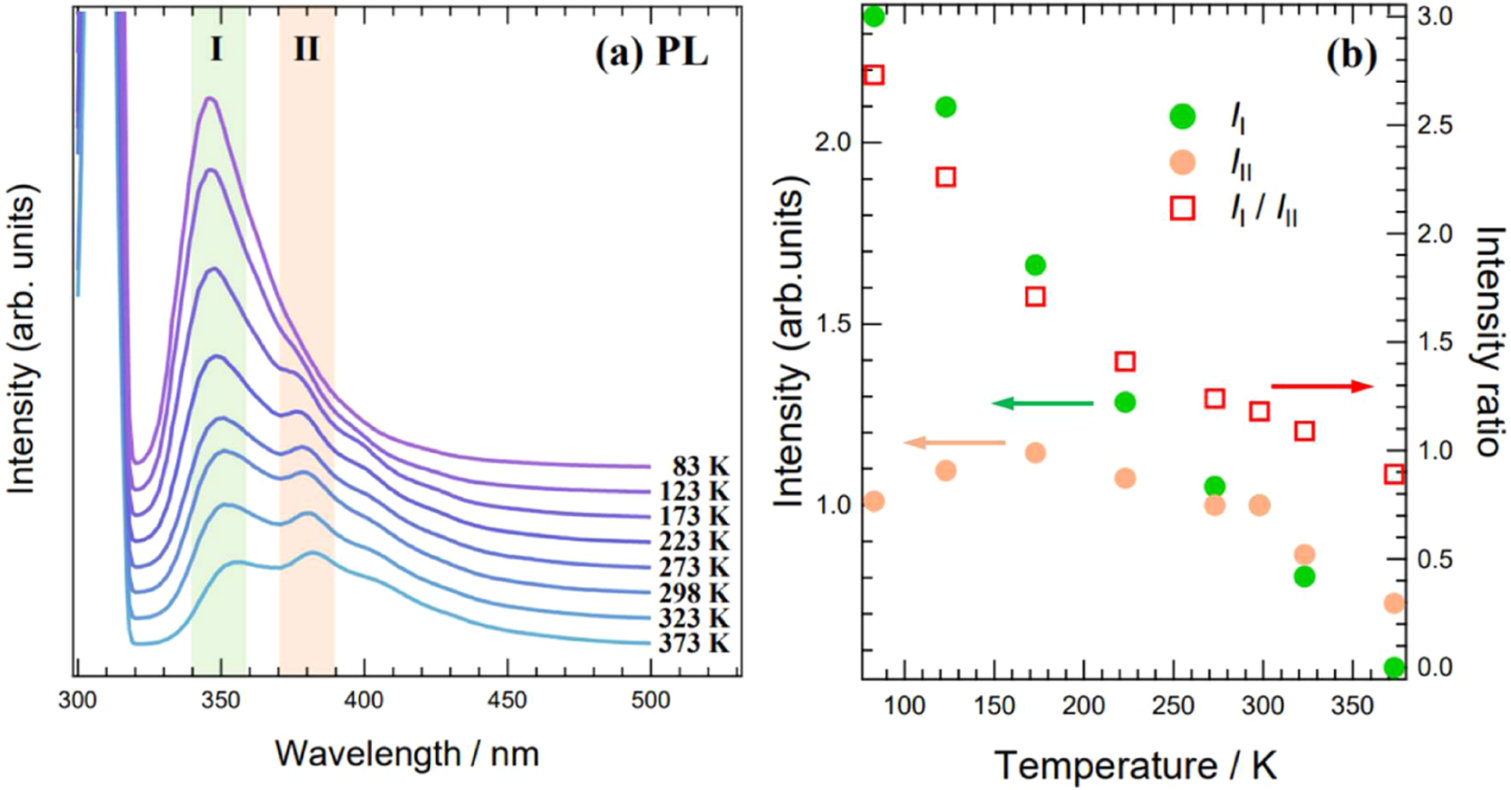
Temperature dependence of the Mhp PL spectrum, measured with a stepwise temperature increase from 298 to 373 K and a stepwise temperature decrease from 298 to 83 K: (a) Mhp PL spectra at each temperature. Horizontal axis is emission wavelength; vertical axis, luminescence intensity. Excitation light wavelength, 300 nm; the strong intensity around 300 nm is the excitation light. (b) Temperature dependence of intensity of two emission peaks (351 nm: *I*_I_, 378 nm: *I*_II_). Horizontal axis, measurement temperature; left vertical axis corresponds to peak intensities *I*_I_ and *I*_II_, right vertical axis to ratio of intensities of the two peaks, *I*_I_/*I*_II_. Both peak intensities are normalized to the value measured at 298 K.

These results confirm that Mhp and Mhp (dehy.), similar to melem, emit in the near-UV region and exhibit high quantum yield and delayed fluorescence. High quantum yields and delayed fluorescence are characteristics of TADF materials. However, to conclude that Mhp exhibits TADF, it is necessary to directly confirm this by measuring the temperature-dependent fluorescence lifetime of Mhp. On the other hand, as shown in Figure 12a, Mhp differs from melem and Mhp (dehy.) because it exhibits multiple emission peaks in the PL spectrum. To investigate these multiple peaks in the PL spectra of Mhp in more detail, we measured the temperature dependence of the Mhp PL spectra. The results are shown in Figure 13.

Figure 13a shows the Mhp PL spectra temperature dependence. The spectrum shape changed with temperature. Among the multiple emission peaks observed at room temperature, the peak at 351 nm is referred to as peak I (green band), and that at 378 nm as peak II (orange band). In addition to peaks I and II, peaks at longer wavelengths were also observed at higher temperatures. However, at lower temperatures, the luminescence intensity of peaks II: *I*_II_ decreased and that of peaks I: *I*_I_ increased. Thus, the luminescence intensities of peaks I and II show different dependences on temperature. We now discuss the detailed temperature dependence of the intensities of peaks I and II. Figure 13b shows the temperature dependence of *I*_I_ and *I*_II_ and the ratio of the intensity of peak I to that of peak II (*I*_I_/*I*_II_). II increased at lower temperatures, reaching an intensity at 83 K approximately 2.3 times that at 298 K. In contrast, *I*_II_ did not increase as markedly as *I*_I_ at lower temperatures, and *I*_II_ decreased at temperatures below 173 K. Consequently, the ratio *I*_I_/*I*_II_ increased with decreasing temperature. Based on the above results on the luminescence intensity temperature dependence, the optical characteristics of Mhp can be summarized as follows: (1) At room temperature, Mhp exhibited multiple emission peaks and emitted light over a wide wavelength range. (2) At low temperatures, the luminescence intensity decreased at longer and increased at shorter wavelengths. Next, the origin of the optical properties of Mhp is discussed in terms of the crystal structure.

First, we discuss the causes of the appearance of multiple emission peaks in the Mhp PL spectrum. In general, the following are possible causes for the appearance of multiple emission peaks.

The first is that the sample was a mixture of several materials. In mixtures of different materials, multiple peaks corresponding to different emission wavelengths are observed. However, when substance mixtures exhibit luminescence at different wavelengths, the PL spectra are often dominated by luminescence from the long-wavelength side,^31,32^ and this hypothesis offers no obvious explanation for the decrease in long-wavelength luminescence intensity at low temperatures. This is because when the sample contains a small amount of impurities, energy transfer from the sample to the impurities occurs, causing luminescence in the long wavelength region. Therefore, this possibility can be ruled out for Mhp. However, strictly speaking, it is necessary to measure the excitation wavelength dependence of the PL spectrum, etc., to reach this conclusion.

Second, both fluorescence and phosphorescence were observed. Phosphorescence usually has a lifetime of a few ms to a few s, and the afterglow can often be confirmed visually. The luminescence lifetime of Mhp observed in Figure 12b was on the order of nanoseconds, and no afterglow was observed. In addition, the PL spectrum observed on a millisecond time scale did not show any luminescence in the near-ultraviolet region. Therefore, phosphorescence was unlikely to be observed. However, it should be noted that in order to completely rule out the possibility of phosphorescence, it is necessary to carry out fluorescence lifetime measurements at low temperatures.

The third possibility is the involvement of a higher excited state. Normally, molecular luminescence is produced by transitions from the S_1_ excited state, but in some molecules, such as azulene and its derivatives, luminescence produced by transitions from the S_2_ excited state has been reported.^33^ In this case, one of the factors that enables luminescence from S_2_ is that the energy difference between S_0_ and S_1_ is equivalent to the energy difference between S_1_ and S_2_. However, melem, a near-ultraviolet luminescent material, does not satisfy this condition because the energy difference between S_0_ and S_1_ is large. From the above discussion, we believe that these three factors do not fully explain the Mhp optical properties. Therefore, other explanations must be explored for the multiple luminescence peaks exhibited by Mhp. Therefore, we investigated how the Mhp crystal structure affects its excited state.

**Figure 14:**
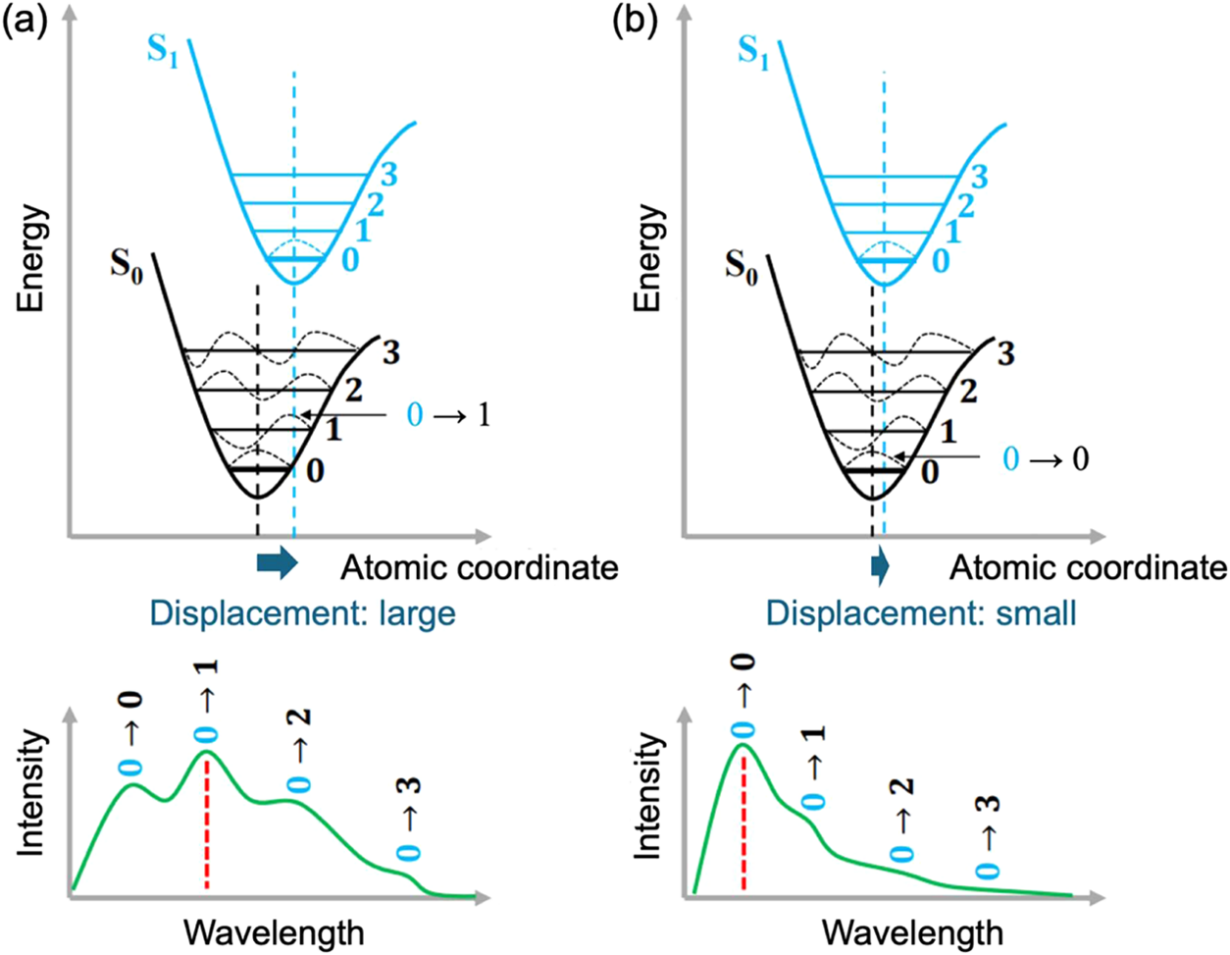
(a) Conceptual diagram of the emission process when the hydrogen bonds with surrounding molecules are weak; (b) conceptual diagram of the emission process when the hydrogen bonds are strong. Horizontal axes show atomic nucleus coordinates; vertical axes show the energy of the molecule. The potential curve drawn in black indicates the potential in the ground state, and that drawn in blue the potential in the excited state. The diagrams below the potential curves show schematic drawings of the emission spectrum produced by each process.

Figure 14 shows the potential energy curves for the molecule vibrational levels with the horizontal axis in atomic coordinates. The black and blue curves represent the potential energies of the ground and excited states, respectively. The minima of the potential curves of the ground and excited states have different atomic coordinates. This indicates that the most stable molecular structures differ in ground and excited states. In addition, according to the Franck–Condon principle, absorption and emission transitions occur perpendicular to the atomic coordinates. Therefore, when a molecule absorbs light, the transition to an excited state occurs perpendicular to the atomic coordinates, followed by relaxation to the most stable structure of the excited state. The excited state then transitions to each vibrational level of the ground state, perpendicular to the atomic coordinates, and emits light. Here, the difference between the molecular structures in the excited state and the ground state is described as “molecular distortion.” Thus, in general, the distortion of excited-state molecules affects the luminescence, the magnitude of which is reflected in the shape of the PL spectrum.

In systems with intermolecular hydrogen bonds, such as Mhp, the degree of molecular distortion in the excited state is likely to depend on the strength of the hydrogen bonds. As shown in Figure 14a, in a system with weak hydrogen bonds, the molecules in excited states are more easily distorted. However, as shown in Figure 14b, in a system with strong hydrogen bonds, the molecules in the excited state are less distorted. Strong hydrogen bonds tend to stabilize the molecular structure. The overlap of the vibrational wave functions of the excited and ground states also changes. Considering the transition probabilities, the greater the overlap between the vibrational wave functions of the excited and ground states, the more likely transitions between states are to occur. As shown in Figure 14a, the displacement between the atomic positions of the excited and ground states is relatively large when the hydrogen bonds are weak, because the molecule is easily distorted. Therefore, the 0 → 1 transition is more likely to occur than the 0 → 0 transition because of the greater overlap between the wave function of the excited state vibrational level ν′ = 1 and the ground state wave function. As a consequence, the luminescence due to the 0 → 1 transition appears strongest in the PL spectrum. In contrast, in the case of strong hydrogen bonds, the overlap between the vibrational wave functions of ν = 0 and ν′ = 0 is the largest, so the luminescence due to the 0 → 0 transition is the strongest in the PL spectra. This is illustrated in Figure 14b. This means that, assuming that hydrogen bond strength varies across a single system, the stronger the hydrogen bond, the stronger the luminescence on the shorter-wavelength side. The characteristics of the PL spectrum of such a system can be summarized as follows: (1) The radiative transitions to the vibrational level of the ground state appear as multiple emission peaks. (2) The luminescence intensity at short wavelengths increases because the excited-state molecular distortion is suppressed due to hydrogen bonding. The characteristics of step (2) also lead to the suppression of nonradiative deactivation. Nonradiative inactivation corresponds to transitions in which the energies of the excited and ground states are equal. In general, the greater the molecular distortion of the excited state, the greater the overlap between the vibrational wave functions of the excited and ground states and the greater the likelihood of nonradiative deactivation. Conversely, in systems with strong hydrogen bonds, if the distortion of the excited-state molecule is small, the overlap of these vibrational wave functions becomes small, such that nonradiative deactivation is less likely to occur. These PL spectral characteristics have also been reported for other molecules, including those with the same heptazine skeleton as melem.^34−38^

It should be stressed that the characteristics of the Mhp PL spectrum shown in Figure 13 are in good agreement with characteristics (1) and (2). But if the same interpretation is to be applied to Mhp, that molecular distortion affects its luminescence properties, the strength of the hydrogen bonds acting on the Mhp melem molecules must be shown to change with temperature. In other words, there must be a factor in the Mhp crystal structure at low temperatures that suppresses the distortion of melem molecules in the excited states. Therefore, single-crystal XRD measurements of Mhp were performed at room temperature and a low temperature (94 K) to investigate the factors that suppress the distortion of excited-state melem molecules.

**Figure 15:**
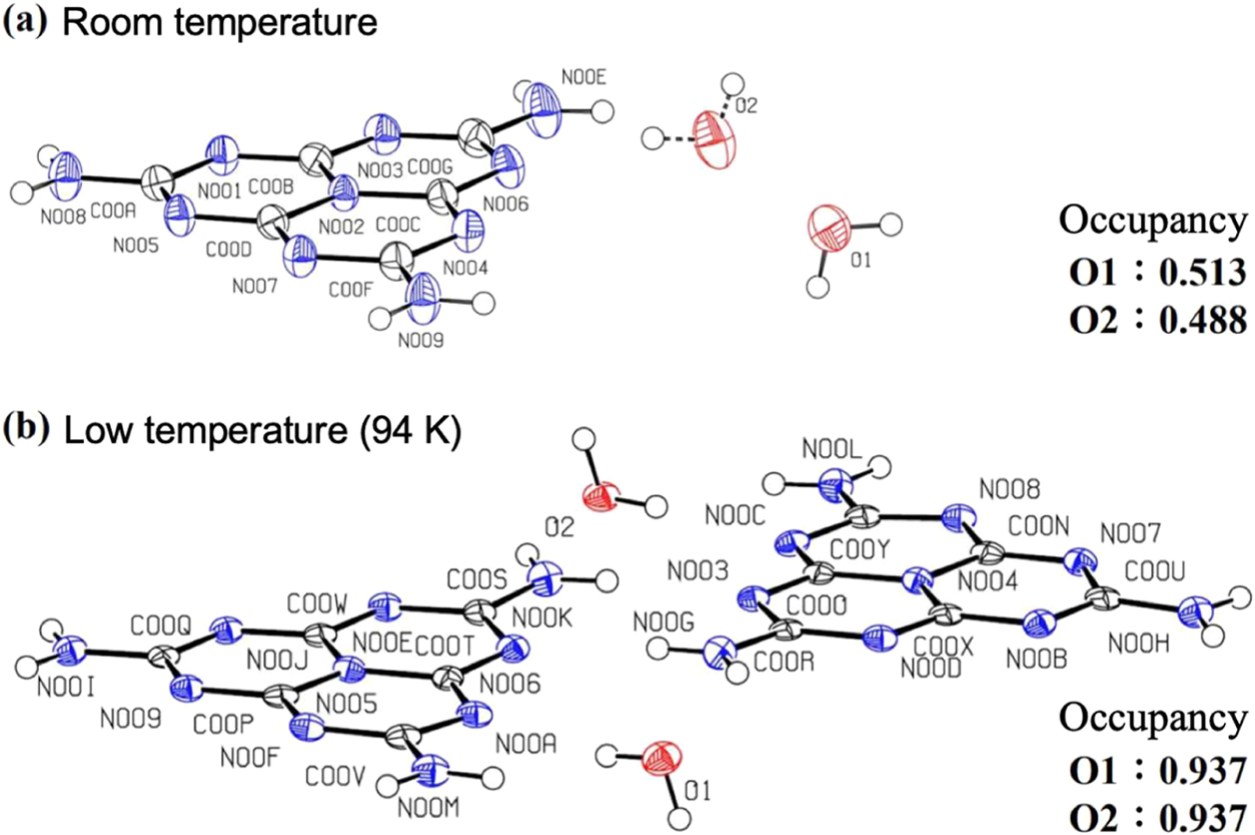
ORTEP drawing of Mhp single crystal obtained from analysis of single-crystal XRD measurements at room temperature (a) and low temperature (94 K) (b). Black, blue, and red ellipsoids represent carbon, nitrogen, and oxygen atoms, respectively. The ellipsoids are isosurfaces with a probability of 50% for the presence of atoms. The small white spheres are hydrogen atoms. PLATON software was used to create the drawing.^21^ The obtained atomic coordinates and occupancies are summarized in Tables S2 and S3.

Figure 15 shows the ORTEP diagrams obtained from single-crystal XRD measurements at room temperature (a) and low temperature (94 K) (b). The room temperature crystal structures are essentially the same as those in Figures 4 and 5, although the measurements were performed on a different sample. The *R* factors for the crystal structures obtained were 5.16% at room and 8.64% at low temperature, so the crystal structures can be considered reasonable. Examining the ellipsoid shapes representing the atoms of the melem molecule in the figure, we saw that at room temperature, they extend in a direction perpendicular to the molecular plane; at low temperatures, they become smaller. The ellipsoids in the ORTEP diagram represent the areas where thermally vibrating atoms exist; therefore, we hypothesize that at low temperatures, the thermal vibration of atoms in a direction perpendicular to the melem molecule is suppressed. Thus, the suppression of thermal vibrations at low temperatures may contribute to the suppression of nonradiative deactivation. Furthermore, the occupancy of the water-molecule oxygen atoms was approximately 0.5 at room temperature, but at low temperatures, it was close to 1.

**Figure 16:**
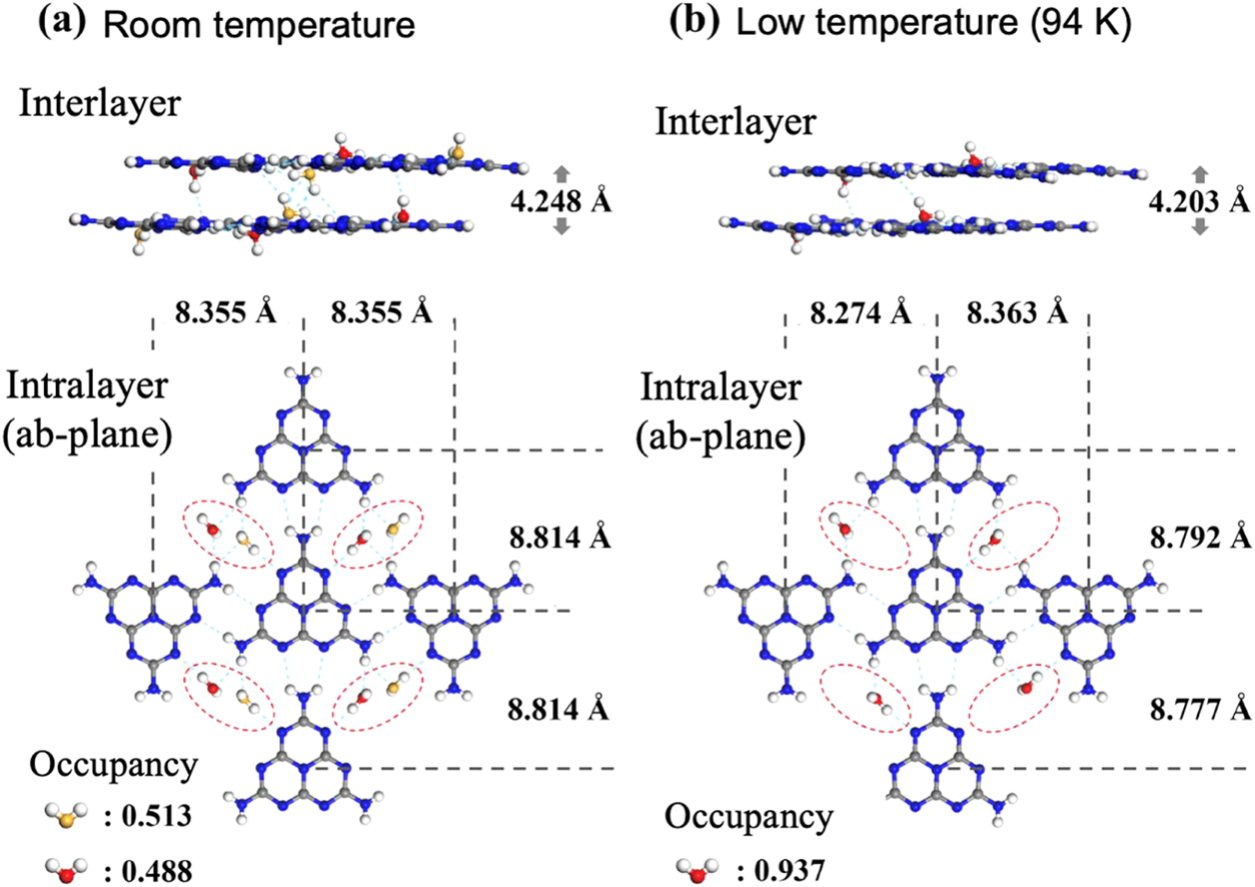
Crystal structure of inter- and intralayers (ab-plane) at (a) room temperature and (b) 94 K as determined by single-crystal XRD. The distance given between molecules in the figure is the distance between the nitrogen atoms at the center of the melem molecules. Light blue dotted lines represent hydrogen bonds.

The crystal structure obtained from single-crystal XRD is shown in Figure 16 to display its changes. Figure 16a,b show the crystal structures of the inter- and intralayers (ab-plane) at room and low temperatures (94 K), respectively. The crystal structures in Figure 16a,b show no structural differences that could significantly affect the arrangement of the melem molecules. However, a change occurs in the arrangement of the water molecules. These are shown enclosed in red dotted circles in the intralayer crystal structure. At room temperature, two adjacent water molecules exist with an occupancy of approximately 0.5 each; however, at low temperatures, only one of them exists with an occupancy of approximately 1. This indicates that the water molecule positions are no longer random at low temperatures, and that the disorder of the water molecules is resolved. Here, we discuss why the disorder of the water molecules in Mhp resolves at low temperatures.

These disorders can be classified into two types. The first is static disorder, in which the atomic positions in each unit cell are random and the atomic positions do not move over time. The second is dynamic disorder. In this disorder, the positions of two atoms move back and forth owing to thermal vibrations, and the time average of these positions results in disorder. Of these two types of disorder, dynamic disorder can sometimes be resolved by lowering the temperature, which suppresses thermal vibration. Because the disorder of the Mhp water molecules was resolved by lowering the temperature, it may possibly have been a dynamic disorder. In this case, the water molecules at room temperature were in a state of large thermal vibrations and were moving back and forth between the two sites. A similar disorder of water molecules has been reported in other hydrates.^39,40^

Next, we examined the arrangement of the melem molecules. The arrangement of melem molecules in Mhp did not change significantly with decreasing temperature, but the distance between the molecules changed. In the interlayer direction, it shortened by about 0.05 Å, and in the intralayer side-to-side and head-to-tail directions, it shortened by about 0.04 and 0.03 Å, respectively. As a result, the hydrogen bond distance between melem molecules decreased by approximately 0.04 Å for both side-to-side and head-to-tail hydrogen bonds. These results confirmed that at low temperatures, the disorder of the Mhp water molecules was resolved, and the hydrogen bond distance between the melem molecules decreased. Therefore, it is reasonable to assume that the decrease in the hydrogen bond length between ground-state melem molecules also contributes to suppression of the melem molecule distortion in the excited state. In addition, the resolution of the water molecule disorder may also contribute to suppression of the melem molecule distortions in the excited states via hydrogen bonds between the melem and water molecules.

From the discussion thus far, the optical properties exhibited by Mhp can be summarized as follows:

(1) Multiple luminescence peaks are seen in the Mhp PL spectrum, originating from vibrational levels in the ground state.
(2) Mhp exhibits decreased hydrogen bond lengths between melem molecules at low temperatures, and the disorder of water molecules is resolved. Consequently, distortion of the melem molecules in the excited states is suppressed and the luminescence intensity at short wavelengths increased.
(3) The suppression of thermal vibrations at low temperatures and of molecular distortion in excited states prevents nonradiative deactivation, resulting in increased luminescence intensity and high fluorescence quantum efficiency. This suppression of molecular distortion in the excited state due to hydrogen bonding represents a possible approach to further shortening the emission wavelengths of NUV-emitting materials and improving their fluorescence quantum efficiency.

## 4 Conclusions

In this study, we determined the crystal structure of melem hydrate, Mhp, and investigated its optical properties.

First, to improve the quality of the Mhp single crystals and increase their size, the growth conditions for Mhp were optimized by controlling the amount of water added. We found that when the ratio of water in DMF to the water mixture used in the ultrasonic treatment increased, the crystal structure shifted gradually from melem crystals to Mhp and then to Mh. Based on these results, a solvothermal method using a mixed solution of DMF and water was devised, and single crystals on the order of 100 μm were successfully grown. These results enabled the growth of samples with sufficient yields and crystal sizes for various physical property measurements.

The crystal structure of Mhp was determined by single-crystal XRD measurements. The results revealed that Mhp is a layered melem hydrate containing disordered water molecules. The structural transition to melem crystals upon heating and dehydration was observed using temperature-dependent XRD measurements. This phase transition of the crystal structure, associated with a decreased amount of water, suggests that hydrogen bonds involving water molecules play an important role in the formation of the Mhp crystal structure. We also successfully grew Mdp, a sample in which the light water of Mhp was replaced with heavy water. FTIR measurements of the Mdp single crystals were carried out using the reflection method, which revealed that the Mdp single crystals exhibited optical anisotropy. This optical anisotropy is due to the orientations of the melem and water molecules in the crystal. Finally, the optical properties of Mhp were evaluated. Similar to melem, Mhp exhibits luminescence in the NUV region of the PL spectrum. This luminescence had a high quantum yield and delayed fluorescence. Furthermore, multiple luminescence peaks were observed in the PL spectrum of Mhp, and the luminescence intensity at short wavelengths increased at low temperatures. To investigate the effect of the crystal structure on optical properties, the temperature dependence of the Mhp crystal structure was investigated in detail using single-crystal XRD measurements. At low temperatures, the disorder of the water molecules in the crystal resolved, and the hydrogen-bond distances between the melem molecules shortened. These results suggest that the multiple emission peaks in the Mhp PL spectra originate from the vibrational levels of the ground state and that the increase in luminescence intensity at low temperatures is caused by the suppression of molecular distortion in the excited state.

The results of this study are expected to provide an important basis for the molecular design of highly efficient luminescent materials based on melem and CN materials. They also provide deep insights into the role of hydrogen bonds in the optical properties of hydrate crystals of similar materials.

## Supporting Information Available

XRD pattern and FTIR spectrum of the synthesized melem. Atomic coordinates and occupancies of each atom of Mhp single crystal at room temperature. Image of the Mhp single crystal used in the single-crystal XRD measurement. Diffraction X-rays mapped onto the reciprocal lattice space, XRD patterns, and TG-DTA results of Mhp and Mdp grown by the solvothermal method. Atomic coordinates and occupancies of each atom of Mhp single crystal at room temperature. Atomic coordinates and occupancies of each atom of Mhp single crystal at 94 K. Calculation method of fluorescence lifetime (PDF)
