# Supplementary material for: Role of Hydrogen Bonding in Crystal Structure and Luminescence Properties of Melem Hydrates
Source: ACS Omega. 2025 Apr 15;10(16):16977–92. doi: 10.1021/acsomega.5c01714 (PMC12044482; doi:10.1021/acsomega.5c01714)
Supplement: Supplementary file 1 — ao5c01714_si_001.pdf [file ao5c01714_si_001.pdf]

Supporting Information

Role of hydrogen bonding in crystal structure and luminescence properties of melem hydrates

Kaname Kanai[1,*], Taiki Yamazaki[1], Hiroki Kiuchi[1], Momoka Isobe[1], Yoriko Sonoda[2]

[1]*Department of Physics and Astronomy, Faculty of Science and Technology, Tokyo University of Science, 2641 Yamazaki, Noda, Chiba 278-8510, Japan*

[2]Research Institute for Advanced Electronics and Photonics, National Institute of Advanced Industrial Science and Technology (AIST), Higashi 1-1-1, 305-8565 Tsukuba, Ibaraki, Japan

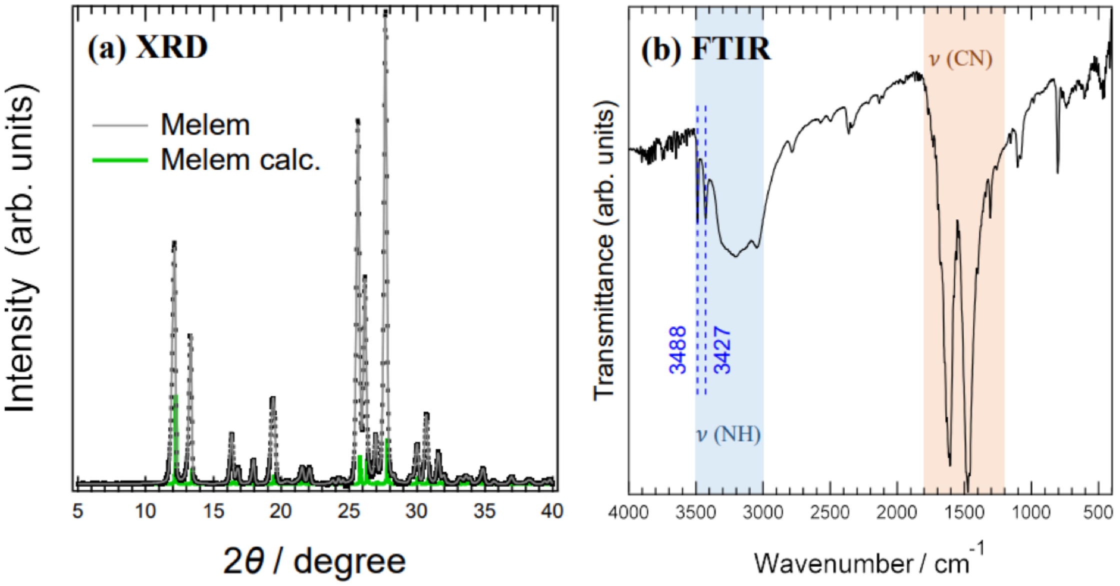

Figure S1. (a) XRD pattern of the synthesized melem. Horizontal axis, diffraction angle 2θ; vertical axis, diffraction intensity. Green line represents simulated XRD pattern of melem crystal (Melem calc.). The simulation was performed using the crystal structure of melem crystal and Mh reported in previous studies.[1] (b) FTIR spectrum of synthesized melem. Horizontal axis, wavenumber; vertical axis, transmittance.

Figure S1(a) shows the XRD patterns of the melem synthesized in this study. The XRD pattern is identical to the XRD pattern of melem crystals reported in a previous study.[1] Figure S1(b) shows the FTIR spectrum of the synthesized melem. The peaks observed in the 1200–1800 cm^{-1} region are derived from the stretching vibration of the CN group of the melem heptazine backbone. The peak observed in the 3000–3500 cm^{-1} region is derived from the stretching vibration of the NH_2 groups at the molecular terminals of melem. In addition, of the peaks in the 3000–3500 cm^{-1} region, the two characteristic peaks at 3427 cm^{-1} and 3488 cm^{-1} are in good agreement with previous studies.[1] Based on the above results, we concluded that melem was also synthesized appropriately in this study, for which Mhp crystals were grown using melem as the precursor.

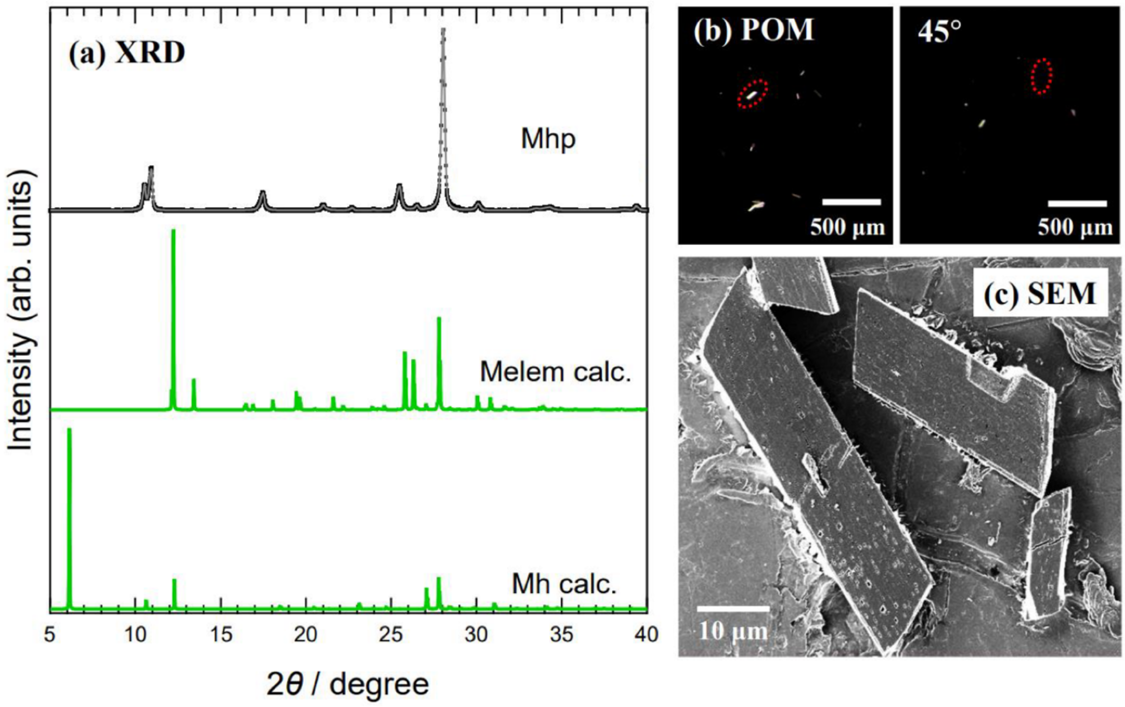

Figure S2. (a) XRD pattern of Mhp grown by vapor diffusion method. Horizontal axis, diffraction angle 2θ; vertical axis, diffraction intensity. Green lines are simulated XRD patterns of melem crystal (Melem calc.) and Mh (Mh calc.). The simulations were performed using the crystal structures of melem crystal and Mh reported in previous studies.[2,3] (b) POM image of Mhp sample. Image on right was taken by rotating sample in image on left by 45°. (c) SEM image of Mhp sample.

Figure S2(a) shows the results of powder XRD measurements of the sample obtained using the vapor diffusion method. When the obtained XRD patterns were compared with the simulated XRD patterns of melem and Mh, it was observed that they differed. In addition, characteristic diffraction peaks at $2\theta = 10.4°$ and 10.8° were observed, consistent with the peaks observed in the XRD pattern of Mhp reported in a previous study.[4] Figure S2(b) shows the POM image of the sample obtained under the crossed-Nicols conditions. Observation of the transmitted light through the sample in this POM image indicated that the sample was crystalline. In addition, when the sample was rotated by 45°, it showed complete extinction, indicating that the sample was uniformly crystalline and a single crystal. Figure S2(c) shows the SEM image of the sample. The sample contained many parallelogram-shaped plate crystals with straight edges (facets). The average size of these crystals was several tens of micrometers.

Table S1. Atomic coordinates and occupancies (Occ.) of each atom of Mhp single crystal at room temperature determined by the crystal structure analysis.

			x	y	z	Occ.
1	N	N001	0.52919	0.81873	0.77355	1.000
2	N	N002	0.52532	0.67737	0.76812	1.000
3	N	N003	0.28840	0.74671	0.54098	1.000
4	N	N004	0.52769	0.53609	0.77294	1.000
5	N	N005	0.75756	0.74973	0.99298	1.000
6	N	N006	0.28528	0.60879	0.54752	1.000
7	N	N007	0.29954	0.88341	0.54974	1.000
8	H	H00A	0.20133	0.88379	0.45353	1.000
9	H	H00B	0.34951	0.92790	0.59798	1.000
10	N	N008	0.76164	0.60478	0.98174	1.000
11	N	N009	0.28918	0.47235	0.56967	1.000
12	H	H00C	0.33765	0.42770	0.61935	1.000
13	H	H00D	0.18834	0.47245	0.47980	1.000
14	C	C00A	0.37361	0.81454	0.62337	1.000
15	N	N00B	0.98252	0.67704	1.19707	1.000
16	H	H00E	1.03354	0.63235	1.24025	1.000
17	H	H00F	1.03094	0.72164	1.24735	1.000
18	C	C00C	0.60673	0.74996	0.84629	1.000
19	C	C00D	0.60699	0.60442	0.84268	1.000
20	C	C00E	0.36335	0.67759	0.61539	1.000
21	C	C00F	0.36932	0.54053	0.63117	1.000
22	C	C00G	0.83000	0.67717	1.05372	1.000
23	O	O1	0.00630	0.37811	0.33120	0.522
24	H	H1A	0.08123	0.34430	0.39702	0.522
25	H	H1B	-0.05927	0.37861	0.39575	0.522
26	O	O2	0.00050	0.47928	0.16170	0.489
27	H	H2A	0.06136	0.47191	0.09241	0.489
28	H	H2B	-0.07536	0.51112	0.08754	0.489

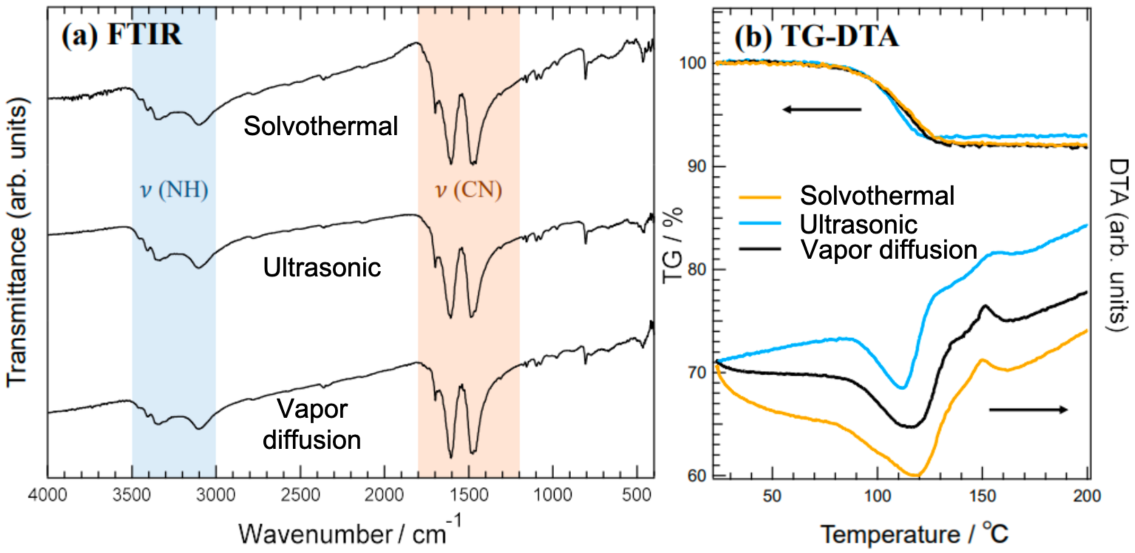

Figure S3. (a)FTIR spectra and (b) TG-DTA measurement results for Mhp samples grown using each method discussed above. FTIR spectrum: horizontal axis, wavenumber; vertical axis, transmittance. TG-DTA graph: horizontal axis, temperature; vertical axis, TG.

Figure S3(a) shows the results of the FTIR measurements of Mhp samples grown using the solvothermal, ultrasonic, or vapor diffusion method. A noticeable absorption was visible in all three 1200–1800 cm^{-1} wavenumber regions (red band). These absorption peaks were attributed to the CN stretching vibration of the melem-molecule heptazine backbone. These results indicate that Mhp molecules produced using any of the three methods retained the melem molecular structure and were not partially broken down by their ultrasonic processing, heating, or high-pressure environment. In addition, significant absorption was observed in all three 3000–3500 cm^{-1} wavenumber regions, and no differences were seen in peak position or intensity across the samples. The absorption peaks in this wavenumber region were attributed to the stretching vibrations of the melem molecule NH_2 groups, as well as the vibrations of the water molecule OH groups. This indicates that no differences in intermolecular hydrogen bonds or hydration-water molecule states existed among the samples.

Figure S3(b) shows the results of TG-DTA measurements of Mhp crystals grown using solvothermal, ultrasonic treatment, or vapor diffusion methods. At temperatures near room temperature, no mass was lost from heating, but near 120 °C, an endothermic

reaction accompanied by mass loss took place. The mass loss was caused by the desorption (dehydration) of water attached to the sample surface or encapsulated in the crystals. From these results, the ratio of water molecules to 1 mol of Mhp melem molecules was estimated to be 1.02 mol (vapor diffusion), 0.94 mol (ultrasonic), and 1.00 mol (solvothermal). The water content of the sample grown using ultrasonic treatment was slightly lower than that of the other samples, but the temperature at which dehydration occurred and the FTIR measurement results were the same for all samples, indicating no difference in the chemical environment of the hydration water molecules in each sample. The molar ratio of water molecules to melem molecules in Mhr reported in previous research was 0.97 mol, which is almost the same as in this study.[4] According to the previous research, the ratio of Mhr is 1.82 mol, which is approximately twice that of Mhp. As can be seen in Figure 1, this indicates that, compared to Mhp, more water molecules are accommodated within the large channels present in the crystal structure of Mhr. These water molecules support the crystal structure of Mhr.

The DTA results of Mhp in Figure S3(b) show that an exoergic reaction was observed at roughly 150 °C, immediately after dehydration, without a decrease in mass. This exoergic reaction corresponded to a crystal structure transition and has been confirmed in previous research.[4]

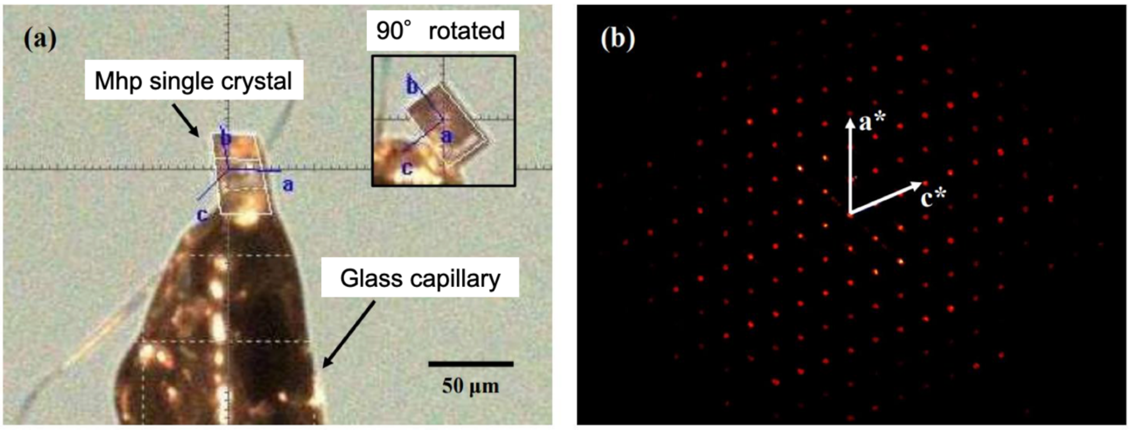

Figure S4. (a) Image of Mhp single crystal used in single-crystal XRD measurements. Mhp crystal was mounted on the tip of a glass capillary. White solid line in figure represents single crystal outline. Single crystal dimensions estimated from image: approximately 25.2 × 29.6 × 40.8 μm^3. (b) Diffraction X-rays mapped onto reciprocal lattice space. Image viewed from downward direction at a^*c^*-plane.

Figure S4(a) shows a photograph of the Mhp sample used for single-crystal XRD measurements. A single crystal of Mhp was fixed to the tip of a glass capillary with a diameter of approximately 100 μm using an instant adhesive. The crystals used for the measurement were selected based on their thicknesses, clear facets, and uniform transmission of light. The axes a, b, and c in the figure represent the crystal axes of the measured sample, as determined from the measurement results. Figure S4(a) shows that the parallelogram faces of the single crystal correspond to the ac-plane of the crystalline structure. Figure S4(b) shows diffraction X-rays mapped onto the reciprocal lattice space. Many periodic red spots are observed; these correspond to reciprocal lattice points. That clear reciprocal lattice points were obtained confirms that the measured sample was a single crystal.

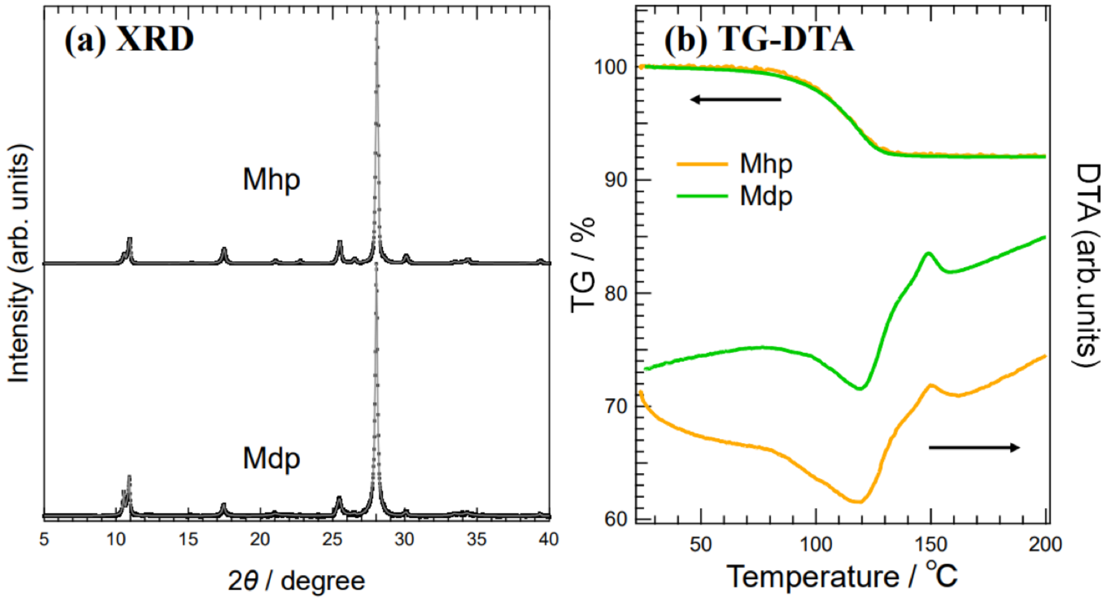

Figure S5. XRD patterns (a) and TG-DTA results (b) of Mhp and Mdp grown by solvothermal method. Horizontal and vertical axes of (a) XRD graph are diffraction angle 2θ and diffraction intensity, respectively. (b) Horizontal axis is temperature; left vertical axis shows sample mass (100% at measurement start). The TG graphs are shown in the upper half of the graph. The right vertical axis shows differential heat, upward pointing to exothermal, downward endothermal. Lower half of graph shows DTA graphs.

Table S2. Atomic coordinates and occupancies (Occ.) of each atom of Mhp single crystal at room temperature determined by the crystal structure analysis.

			X	y	z	Occ.
1	N	N001	0.52919	0.18132	0.77350	1.000
2	N	N002	0.52523	0.32283	0.76810	1.000
3	N	N003	0.75763	0.25051	0.99290	1.000
4	N	N004	0.52775	0.46399	0.77310	1.000
5	N	N005	0.28861	0.25328	0.54160	1.000
6	N	N006	0.76150	0.39528	0.98180	1.000
7	N	N007	0.28521	0.39126	0.54760	1.000
8	N	N008	0.29939	0.11668	0.55030	1.000
9	H	H00A	0.20099	0.11617	0.45455	1.000
10	H	H00B	0.34962	0.07231	0.59866	1.000
11	N	N009	0.28913	0.52770	0.56950	1.000
12	H	H00C	0.33763	0.57229	0.61922	1.000
13	H	H00D	0.18831	0.52762	0.47957	1.000
14	C	C00A	0.37320	0.18545	0.62310	1.000
15	C	C00B	0.60620	0.25026	0.84580	1.000
16	C	C00C	0.60684	0.39586	0.84230	1.000
17	C	C00D	0.36263	0.32257	0.61520	1.000
18	N	N00E	0.98248	0.32304	1.19730	1.000
19	H	H00E	1.03093	0.27847	1.24729	1.000
20	H	H00F	1.03343	0.36766	1.24081	1.000
21	C	C00F	0.36910	0.45970	0.63090	1.000
22	C	C00G	0.82980	0.32297	1.05370	1.000
23	O	O2	0.99990	0.52089	1.16200	0.488
24	H	H2A	1.06289	0.53230	1.09780	0.488
25	H	H2B	0.93199	0.48649	1.08522	0.488
26	O	O1	1.00660	0.62187	1.33100	0.513
27	H	H1A	0.94543	0.61680	1.40074	0.513
28	H	H1B	1.07627	0.65812	1.39417	0.513

Table S3. Atomic coordinates and occupancies (Occ.) of each atom of Mhp single crystal at 94 K determined by the crystal structure analysis.

			x	y	z	Occ.
1	N	N003	0.74420	0.68253	0.51668	1.000
2	N	N004	0.74560	0.82440	0.51291	1.000
3	N	N005	0.75290	0.32174	0.48730	1.000
4	N	N006	0.75900	0.46309	0.48476	1.000
5	N	N007	0.74210	0.96613	0.51222	1.000
6	N	N008	0.64870	0.89816	0.63048	1.000
7	N	N009	0.75700	0.17985	0.48707	1.000
8	N	N00A	0.84550	0.39255	0.36753	1.000
9	N	N00B	0.84740	0.89175	0.39198	1.000
10	N	N00C	0.63660	0.75332	0.63048	1.000
11	N	N00D	0.85530	0.75345	0.39518	1.000
12	N	N00E	0.65200	0.39178	0.60717	1.000
13	N	N00F	0.85960	0.24763	0.37130	1.000
14	N	N00G	0.85400	0.61594	0.40271	1.000
15	H	H00A	0.90239	0.61467	0.35231	1.000
16	H	H00B	0.83041	0.57073	0.42926	1.000
17	N	N00H	0.82700	1.02845	0.39122	1.000
18	H	H00C	0.80067	1.07487	0.41488	1.000
19	H	H00D	0.86785	1.02712	0.33952	1.000
20	N	N00I	0.64820	0.11595	0.60361	1.000
21	H	H00G	0.60079	0.11619	0.65421	1.000
22	H	H00H	0.67215	0.06997	0.57859	1.000
23	N	N00J	0.64340	0.25345	0.60685	1.000
24	N	N00K	0.68040	0.52885	0.60434	1.000
25	H	H00I	0.70889	0.57415	0.57894	1.000
26	H	H00J	0.64076	0.52968	0.65625	1.000
27	N	N00L	0.53900	0.82721	0.74164	1.000
28	H	H00E	0.52005	0.87336	0.76718	1.000
29	H	H00F	0.51124	0.78170	0.76681	1.000
30	N	N00M	0.94590	0.31868	0.25672	1.000
31	H	H00K	0.97189	0.27267	0.23217	1.000
32	H	H00L	0.96257	0.36429	0.22985	1.000
33	C	C00N	0.71080	0.89835	0.55286	1.000

34	C	C00O	0.70970	0.75222	0.55475	1.000
35	C	C00P	0.78970	0.24881	0.44724	1.000
36	C	C00Q	0.68340	0.18487	0.56506	1.000
37	C	C00R	0.81640	0.68607	0.43868	1.000
38	C	C00S	0.69710	0.45964	0.56459	1.000
39	C	C00T	0.78560	0.39424	0.44542	1.000
40	C	C00U	0.80510	0.96082	0.43328	1.000
41	C	C00V	0.88190	0.31928	0.33376	1.000
42	C	C00W	0.68060	0.32236	0.56869	1.000
43	C	C00X	0.81860	0.82309	0.43169	1.000
44	C	C00Y	0.60880	0.82627	0.66592	1.000
45	O	O1	0.91230	0.51957	0.25095	0.937
46	H	H1A	0.81750	0.52901	0.21501	0.937
47	H	H1B	0.86624	0.48350	0.28449	0.937
48	O	O2	0.57980	0.62277	0.74646	0.937
49	H	H2A	0.68833	0.60997	0.77253	0.937
50	H	H2B	0.61311	0.65841	0.70963	0.937

Calculation method of fluorescence lifetime

The lifetime τ of the fluorescence produced by a single transition process is expressed by Equation 1, where the luminescence intensity at t = 0 s is A_0, and the intensity after t seconds is $I(t)$.

$$I(t) = A_0 \exp\left(-\frac{t}{\tau}\right) \quad (1)$$

Because the total fluorescence intensity is influenced by various transition processes, it can be considered as a sum of single-emission components with different fluorescence lifetimes. Therefore, the intensity of n luminescent components can be written as Equation 2.

$$I(t) = \sum_{i=1}^{n} A_i \exp\left(-\frac{t}{\tau_i}\right) \quad (2)$$

From Equation 2, the graph of fluorescence lifetime with logarithmic vertical axis is represented by the superposition of n lines with different slopes. In Figure 13(b), the slope of the graph also changes, showing both prompt fluorescence with a short lifetime and delayed fluorescence with a long lifetime. Therefore, using Equation (2), we performed a fitting analysis of the fluorescence lifetime graph in Figure 13(b), assuming the lifetime consisted of two components. The results of this analysis are shown in Table 2. The average fluorescence lifetime τ was calculated using Equation 3.[5]

$$\tau = \frac{A_1\tau_1^2 + A_2\tau_2^2}{A_1\tau_1 + A_2\tau_2} \quad (3)$$

References

(1) Jürgens, B.; Irran, E.; Senker, J.; Kroll, P.; Müller, H.; Schnick, W., Melem (2,5,8-Triamino-tri-s-triazine), an Important Intermediate during Condensation of Melamine Rings to Graphitic Carbon Nitride: Synthesis, Structure Determination by X-ray Powder Diffractometry, Solid-State NMR, and Theoretical Studies. *J Am Chem Soc* **2003**, *125*, 10288– 10300, DOI: 10.1021/ja0357689

(2) Makowski, S. J.; Köstler, P.; Schnick, W., Formation of a Hydrogen-Bonded Heptazine Framework by Self-Assembly of Melem into a Hexagonal Channel Structure, Chem. - A Euro. J., **2012**, *18*, 3248–3257, DOI: 10.1002/chem.201103527

(3) Jürgens, B.; Irran, E.; Senker, J.; Kroll, P.; Müller, H.; Schnick, W., Melem (2,5,8-Triamino-tri-s-triazine), an Important Intermediate during Condensation of Melamine Rings to Graphitic Carbon Nitride: Synthesis, Structure Determination by X-ray Powder Diffractometry, Solid-State NMR, and Theoretical Studies, *J. Am. Chem. Soc.,* **2003**, *125*, 10288–10300, DOI: 10.1021/ja0357689

(4) Dai, T.; Kiuchi, H.; Minamide, H.; Miyake, Y.; Inoki, H.; Sonoda, Y.; Tsutsumi, J.; Kanai, K., Growth and characterization of melem hydrate crystals with a hydrogen-bonded heptazine framework, Phys. Chem. Chem. Phys., **2022**, *24*, 13922–13934, DOI: 10.1039/D2CP00691J

(5) Berezin, M. Y.; Achilefu, S., Fluorescence Lifetime Measurements and Biological Imaging, Chem. Rev., **2010**, 110, 2641–2684, DOI: 10.1021/cr900343z
